# Improving DOT reconstruction accuracy without MRI: Practical guidelines from systematic head model evaluations

**DOI:** 10.1162/IMAG.a.1445

**Published:** 2026-11-12

**Authors:** Nils Harmening, David A. Boas, Alexander von Lühmann

**Affiliations:** BIFOLD – Berlin Institute for the Foundations of Learning and Data, Berlin, Germany; Intelligent Biomedical Sensing (IBS) Lab, Machine Learning Department, Technical University of Berlin, Berlin, Germany; Boston University, Neurophotonics Center, Department of Biomedical Engineering, Boston, MA, United States

**Keywords:** fNIRS, DOT, inverse problem, source reconstruction, individualized head models, photogrammetry

## Abstract

High-density diffuse optical tomography (HD-DOT) is a promising modality for functional brain imaging, usable as a complement or an alternative to fMRI. Head models are commonly built from participant-specific MRI scans, but MRI access is often exactly what is missing in the settings where HD-DOT is most useful. We, therefore, systematically evaluated localization accuracy across a range of head models—subject-specific MRI-based, photogrammetry-based individualized, and atlas-based generic models—using simulated diffuse optical tomography (DOT) data from 15 participants. Our results demonstrate that individualized head models derived from photogrammetry scans lower the median peak localization error by about 3.7 mm relative to the atlas models at every tested probe density, and narrow the reconstructed extent by 7.6 mm at the sparse probe down to 3.1 mm at high density. Across the four tested levels, the spatially variant regularization parameter moves the median peak error by up to 7 mm, comparable with the difference between the best and the worst head model. Its optimum is more stable as a rank in the probe’s sensitivity distribution than as a fixed sensitivity value. We further analyze how localization accuracy relates to probe density and cortical region. By offering a comprehensive resource that maps reconstruction performance onto standard MNI coordinates and cortical parcels, this work delivers practical guidelines for selecting optimal head modeling strategies in HD-DOT studies when subject-specific MRIs are not available.

## 1 Introduction

Functional near-infrared spectroscopy (fNIRS) is a non-invasive neuroimaging technique that measures hemodynamic changes in the brain to infer neural activity (Scholkmann et al., 2014; Yücel et al., 2021). Its tomographic extension, diffuse optical tomography (DOT), enables spatial reconstructions of the brain’s functional activity over time (D. Boas et al., 2001; D. A. Boas et al., 2004). DOT reconstructs cortical activity in source space rather than in channel space, which brings its spatial specificity close to fMRI over the cortex it reaches (Eggebrecht et al., 2012; Wheelock et al., 2019). It does so with wearable hardware that moves with the participant (Chekin et al., 2025; O’Brien et al., 2024; von Lühmann et al., 2024), which allows experiments in settings and with populations that an MRI scanner excludes.

Accurate DOT image reconstruction relies on simulating photon transport through the different biological tissues of the human head. Recent studies have shown that the anatomical accuracy of these head models is critical to reconstruction quality (Srinivasan et al., 2024; Tran et al., 2020; Zhan et al., 2012).

However, creating subject-specific head models has conventionally required a structural MRI scan of every participant—an acquisition that is often too costly, time consuming, and logistically challenging. As a result, most fNIRS and DOT studies still rely on standardized anatomical templates such as Colin27 or the ICBM-152 atlas, which can introduce significant localization errors.

Several studies have already tackled the problem of quantifying this tradeoff. Cooper et al. (2012) reported average localization errors of approximately 18 mm in Euclidean space using Colin27 versus 9.1 mm when using the subject’s individual MRIs. Ferradal et al. (2014) demonstrated that for visual evoked responses, atlas-based reconstructions on the ICBM-152 differ by 2.7 mm from individual MRI reconstructions and by 6.6 mm from fMRI. More recently, Srinivasan et al. (2024) showed substantial variability in parcel sensitivity across subjects in the prefrontal, motor, and visual cortices using atlas-based models, linking it to the difference between generic and subject-specific optode positions, whose median reached 27.4 mm over the highest error motor cortex parts and 16.3 mm prefrontally, against 5.9 mm over the visual cortex. They obtained the subject-specific positions from photogrammetry.

However, prior work often averages over multiple brain regions, restricts analysis to only a few brain regions (Ferradal et al., 2014; Srinivasan et al., 2024), or limits analysis to superficial cortical activations (Cooper et al., 2012). Also, prior studies do not all account for noise or for modern inversion techniques during sensitivity matrix inversion, such as spatially variant regularization (SVR).

Importantly, while photogrammetry is increasingly used for accurate optode digitization and improves both sensor localization and its registration to a head atlas (Mazzonetto et al., 2022), the potential of individualized head models derived from scalp measurements, such as photogrammetric scans, remains underexplored in the DOT context.

Much of the field’s most demanding work already relies on generic head models. Stimulus decoding from HD-DOT was reconstructed on the ICBM-152 atlas (Tripathy et al., 2020), published naturalistic-viewing data are reconstructed on an atlas (Sherafati et al., 2025), the wearable infant benchmark of Frijia et al. (2021) uses an atlas-derived head model, and whole-head infant mapping uses an age-matched template scaled to each infant’s head measurements (Collins-Jones et al., 2024). Work pushing decoding accuracy further has gone the other way and builds the forward model from each participant’s own MRI (Markow et al., 2025). An MRI-free pipeline aims to bring part of that accuracy to the studies that cannot acquire one. Where an MRI is hard to obtain, especially for young children, a generic model has been the only option. Improving the MRI-free head model, therefore, matters across much of the field.

Photogrammetry-based individualization is a promising solution between atlas and MRI. It approximates the individual head anatomy by warping template brains to the measured scalp surface or by using a large database of head anatomies. Individualized head models thus improve anatomical accuracy without requiring full MRI scans. Our previous work demonstrated that individualized head models enhance EEG source localization accuracy (Harmening et al., 2026). These head models, moreover, preserve the advantage of group analysis by mapping into the common MNI coordinate space while reducing reconstruction bias. Exploring the potential to improve image reconstruction performance through head model individualization becomes particularly relevant for HD-DOT acquisitions that, given accurate head models, can achieve fMRI-like resolutions (Eggebrecht et al., 2012; Habermehl et al., 2012).

Therefore, we revisit the comparison of subject-specific and atlas anatomies and include individualized head models from photogrammetry scans, extending and unifying prior efforts to address a key question for the DOT community:

**With or without MRI, which head models minimize localization errors in MNI space given a certain probe sparsity and region of interest (ROI)?**

To facilitate practical decision making in HD-DOT studies, this work provides a systematic resource that enables researchers to select the optimal head modeling strategy (derived from subject-specific MRI, atlas-based, or photogrammetry-based individualization methods), based on their specific probe configurations and targeted cortical regions of interest, all referenced to standard MNI space and cortical parcels.

We evaluate localization accuracy across 1000 parcels from the Schaefer2018 atlas in 15 individuals. We further analyze the influence of probe density, signal depth, and SVR parameters. Our goal is to provide practical guidelines for maximizing localization accuracy in real-world DOT applications.

## 2 Methods

### 2.1 Data collection

We acquired a structural MRI scan of each of 15 subjects (11 male, 4 female, aged 23 to 59, mean 35 ± 8 years) and scanned the outer head shape with a *Shining 3D EinStar 3D-Scanner*. All experiments involving humans were conducted with approval from the ethics committee of the TU Berlin, following the Declaration of Helsinki. All subjects gave written informed consent. The photogrammetry workflow is described in detail below and closely follows the procedure of optode digitization in this video recording for one subject^1^.

- Measure the subject’s head circumference from NAS via RPA, inion (Iz), LPA, and back to NAS with a flexible measuring tape. Choose a cap from the closest cap size (54 cm, 56 cm, 58 cm, 60 cm), adapting downward, and attach it to the subject without any optodes or (spring) holders. Ensure that the cap is fitted tightly enough to minimize hair convexities.
- Scan the subject’s head following the instructions of the *Shining 3D* recording software. The scans took approximately 2–5 minutes each and the subject was instructed to sit still. Export the scan as .obj file.
- In a mesh manipulation software such as MeshLab^2^, manually crop the mesh so it only contains the cap and head without everything below the ears and resave the cropped mesh as an .obj file.
- Load and plot the cropped mesh in the cedalion toolbox (Middell et al., 2026) and manually detect the fiducials (NAS, LPA, RPA) or read their coordinates out if having marked them on the subject before scanning.

The resulting mesh vertices and fiducials were loaded into cedalion, transformed into the CTF coordinate system, cut above the ears (35 mm above the plane spanned by the connecting vectors between NAS, LPA, and RPA), decimated to around 150 vertices and used as a scalp surface proxy, since around 150 well-distributed points are a good choice for the individualization fits (Harmening et al., 2026).

### 2.2 Head model construction

Seven different head models are generated for each of the 15 subjects to evaluate their performance in image reconstruction and compare them with the original MRI-based head model. We distinguish between the following three head model groups:

- MRI-based **individual**,
- photogrammetry-based **individualized**,
- atlas-based **generic**.

All meshes were transformed into binary masks for each tissue in NIfTI format as the output after segmentation with SPM12, using our pipeline, which is described in more detail below.

#### 2.2.1 MRI-based individual head models

The following head models were obtained by segmenting the T1-weighted MRI scans of the individual subjects. To segment the MRI scans as similarly as possible, the automatic segmentation pipeline by Harmening and Miklody (2022) was used. In brief, it includes the following steps: Transforming the MRI scans into the ACPC coordinate system, segmentation using SPM12, post-processing of segmentation masks, volume mesh generation, and surface mesh generation. It is schematically shown in the upper stream of Figure 1. The following head models are generated:

**Fig. 1.**
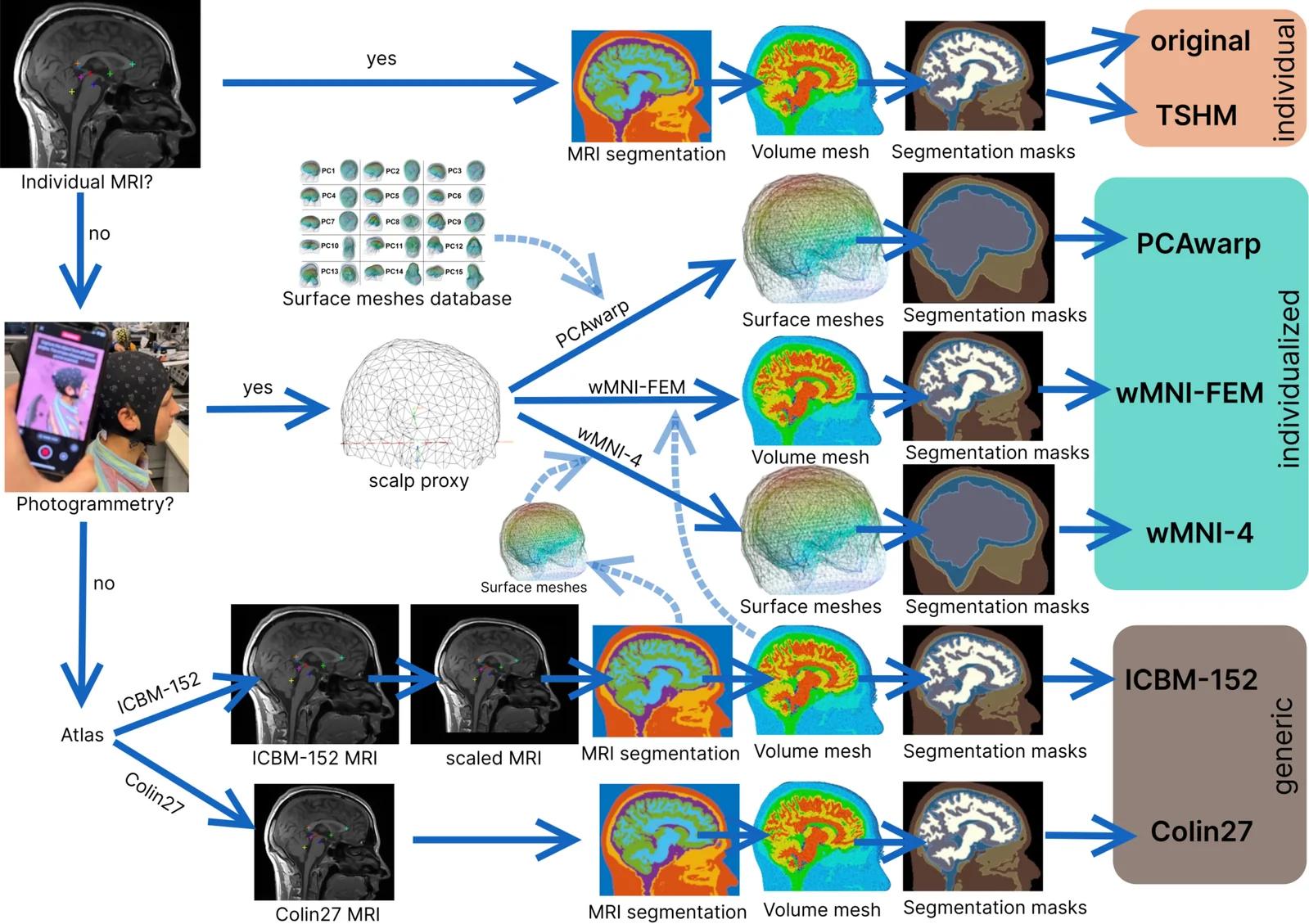
Which head model to choose? Creation pipeline for all head models, depending on whether MRI or photogrammetry is available. The individual head models (original, TSHM) are MRI based, the individualized head models (PCAwarp, wMNI-FEM, wMNI-4) are photogrammetry based, and the generic head models (ICBM-152, Colin27) are atlas based. The outputs for all streams are segmentation masks that can be fed into cedalion.

**Original:** A volume mesh derived from the subject’s MRI scan containing the scalp, skull, cerebrospinal fluid (CSF), and gray and white matter. This head model is used as the ground truth and is expected to perform best in source reconstruction.

**TSHM:** The TwoSurfaceHeadModel is derived from the subject’s own MRI scan, which contains the scalp, skull, CSF, and gray and white matter. After the photon simulation, the system size is reduced by introducing a simplification that maps all brain activity to the cortical surface. This head model is thus restricted to localizing activity on the cortical surface and is commonly used in AtlasViewer (Aasted et al., 2015) and cedalion. We expect this head model to perform similarly to the original head model but to pull the source reconstruction from deeper regions to the cortical surface.

#### 2.2.2 Photogrammetry-based individualized head models

The following head models are generated from the scalp proxies of the photogrammetry scans, schematically shown in the middle stream of Figure 1. Two of them (PCAwarp and wMNI-4) only approximate the different biological tissue surfaces and do not attempt to approximate the details of gyri and sulci. We expect the individualized head models to benefit from having a more similar anatomy to that of the individual and outperform the generic head models in source reconstruction.

**PCAwarp:** A surface mesh head model is derived from the PCAwarp individualization algorithm based on a large head model database (Harmening et al., 2026) with the smoothed cortex surface commonly used in BEM head models for EEG source reconstruction.

**wMNI-FEM:** A volume mesh head model is derived from an extension of the wMNI-4 individualization algorithm (Akalin Acar & Makeig, 2013; Darvas et al., 2006). The scalp surface mesh of the average ICBM-152 head (V. Fonov et al., 2011; V. S. Fonov et al., 2009) was warped to the scalp proxy and the warping transformation was applied to the volume mesh head model of the ICBM-152 head.

**wMNI-4:** A surface mesh head model is derived from the wMNI-4 individualization algorithm as implemented in the EEG Neuroelectromagnetic Forward Head Modeling Toolbox (NFT) (Acar & Makeig, 2010). The scalp surface mesh of the average ICBM-152 head was warped to the scalp proxy and the warping transformation was applied to the remaining surface meshes (skull, CSF, cortex) of the ICBM-152 head.

#### 2.2.3 Atlas-based generic head models

For comparison, the following generic head models are used, segmented with the same pipeline as the MRI-based head models (see Fig. 1 bottom stream):

**ICBM-152:** A volume mesh head model is derived from the MNI ICBM-152 head model, scaled per subject to the individual anatomical landmarks. As a cross-subject atlas, we expect the ICBM-152 to outperform Colin27 in source reconstruction.

**Colin27:** An unscaled volume mesh head model is derived from the Colin27 head model (Holmes et al., 1998). Since it is a head model of a single individual, we expect Colin27 to perform overall worst in source reconstruction.

### 2.3 Probe design

#### 2.3.1 Optode densities

Three whole-head fNIRS/DOT montages of different probe densities were chosen:

- A sparse whole-head fNIRS probe with 34 sources and 32 detectors, which is a modified version of the default NSP2 32 x 32 configuration to expand over the central line. The 112 channels (1st neighbors) have a median distance of (36 ± 4) mm.
- A medium-density ninjaNIRS montage of hexagonal layout with 58 sources and 144 detectors (O’Brien et al., 2024), where 2 sources were added to close a gap over the central line. The 592 channels (1st, 2nd, 3rd neighbors) have median distances of (19 ± 2) mm, (33 ± 2) mm and (40 ± 2) mm.
- A high-density ninjaNIRS montage of hexagonal layout with 165 sources and 496 detectors. The 4404 channels (1st, 2nd, 3rd, 4th, 5th neighbors) have median distances of (12 ± 1) mm, (21 ± 1) mm, (24 ± 2) mm, (32 ± 2) mm, and (40 ± 1) mm.

It should be pointed out that other valid alternatives for DOT probe geometry and density exist (e.g., grid layouts as in Fogarty et al., 2025; White & Culver, 2010), which can result in different sensitivity distributions. In this study, we utilize a set of three probe layouts to facilitate a quantitative and qualitative comparison between head models, enabling the reader to qualitatively transfer our findings to their own probe layout and target ROI.

The probes aligned to an example subject and their corresponding sensitivity profile on the brain surface is shown in Figure 2.

**Fig. 2.**
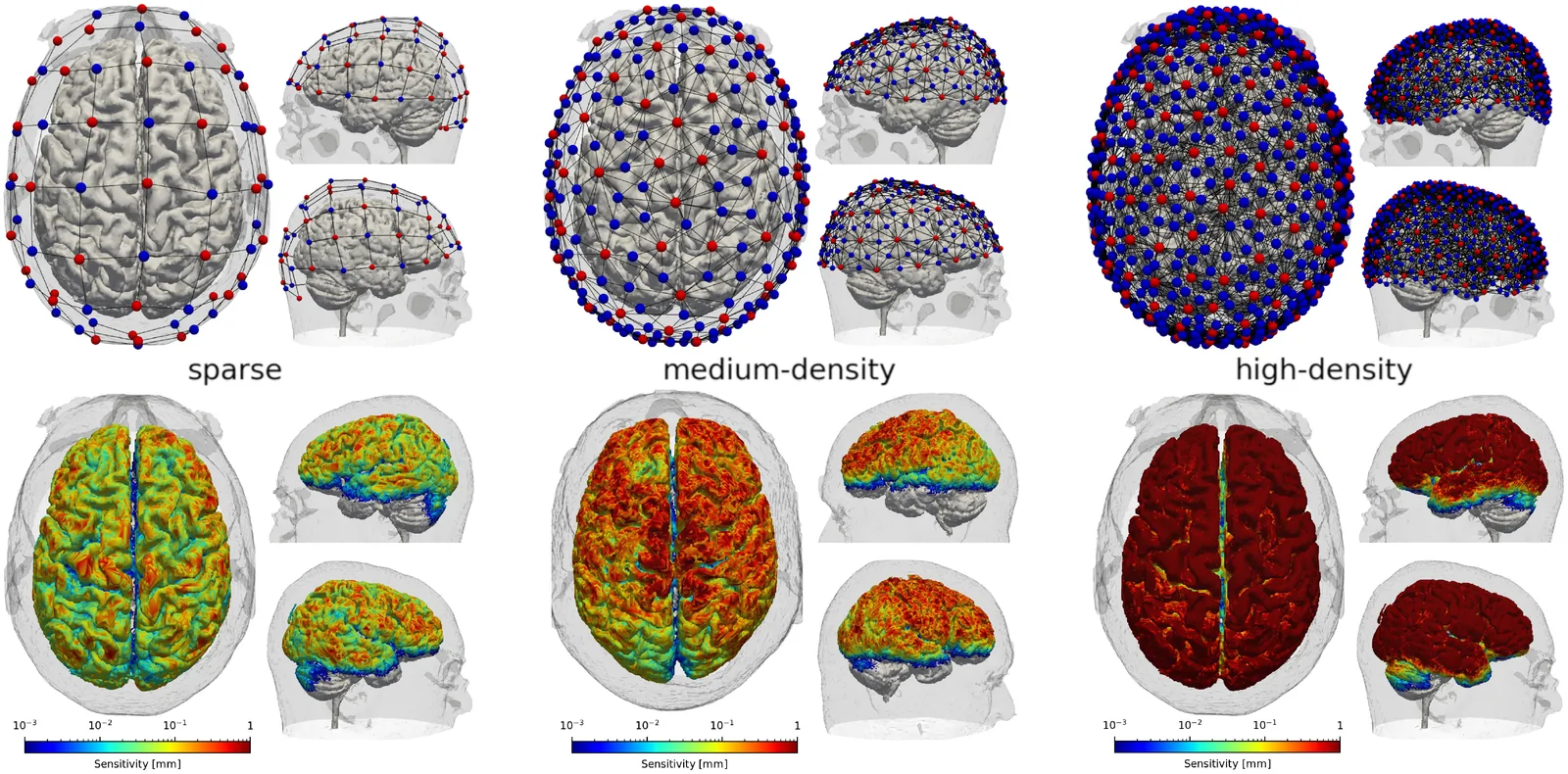
The sparse (top left), medium-density (top center), and high-density (top right) probe montages on an example subject with sources (red dots), detectors (blue dots), and channels (black line). At the bottom, the sensitivity profiles as explained in Section 2.4 for each probe are shown. For visibility, the voxel sensitivities are summed up to the closest cortex surface vertex as in the TSHM.

#### 2.3.2 Optode alignment

To align the optode positions thoroughly but automatically to every individual head model, the individual fiducials NAS, RPA, Iz, and LPA were manually picked. Using the anatomical landmarks, we additionally computed the EEG-1010-system landmarks by using cedalion’s *LandmarksBuilder*. The intersection of 79 landmarks with the landmarks provided by the probes was used to compute the affine transformation, which was then applied to the optodes to align them with the head model. The aligned optodes were then snapped to the scalp surface and its surface normals used as optode directions.

### 2.4 Photon simulation

The relationship of how fluctuations in measured infrared light at the optode channels relate to temporal changes in the optical properties within the tissue volume is typically expressed as

y=Ax+n. (1)

Here, y represents the vector of surface measurements of change in optical density (unitless) obtained from various source–detector pairs (channels), n is the measurement noise vector, and x denotes the changes in absorption properties (in mm^−1^) within the volume. A is the sensitivity matrix (in mm), which maps small changes in internal optical properties to corresponding changes in surface measurements.

As forward model we used the Monte Carlo eXtreme (MCX) (Fang & Boas, 2009) to run photon simulations of 10^6^ photons for all head models of all subjects with the default tissue properties of cedalion v26.5.1. Following the adjoint Monte Carlo approach (D. A. Boas & Dale, 2005), the sensitivity of the channel formed by source *s* and detector *d* to a cortex voxel *v* is

$$A_{sd,v}=\frac{\varphi_{s}\left({\text{r}}_{v}\right)\varphi_{d}\left({\text{r}}_{v}\right)}{\frac{1}{2}\left[\varphi_{s}\left({\text{r}}_{d}\right)+\varphi_{d}\left({\text{r}}_{s}\right)\right]}\Delta V,$$ (2)

where ϕ*_s_* and ϕ*_d_* (in mm^−2^) are the fluences simulated from the source and from the detector (the latter treated as a source by reciprocity), the denominator is the fluence of each optode at the other one and thus the light the channel measures, and Δ*V* = 1 mm^3^ is the voxel volume. The ratio is in mm^−2^, so each entry of A is in millimeters and gives the sensitivity of one cortex voxel to one channel.

### 2.5 Cortex voxel statistics

For every cortex voxel of the 1000 parcels of the Schaefer2018 atlas (Schaefer et al., 2018), additional information was collected including the sensitivity of the voxel to the probe, the distance to the scalp surface, and the parcel according to the Schaefer2018 atlas.

Based on the inverse MNI deformation field, as gained from the MRI segmentation using SPM (Friston et al., 2007), which gives for each voxel its coordinates in MNI space, we converted every individual brain voxel into MNI coordinates. We looked up the corresponding label using the Schaefer2018 parcellation atlas. The Schaefer2018 atlas is a parcellation of 1000 parcels grouped into the following 17 functional networks (Schaefer et al., 2018; Yeo et al., 2011), as plotted in Figure 3: Visual A (VisCent), Visual B (VisPeri), Somatomotor A (SomMotA), Somatomotor B (SomMotB), Dorsal Attention A (DorsAttnA), Dorsal Attention B (DorsAttnB), Salience A (SalA), Salience B (SalB), Control A (ContA), Control B (ContB), Control C (ContC), Default A (DefaultA), Default B (DefaultB), Default C (DefaultC), Temporal Parietal (TempPar), Limbic A (LimbicA), and Limbic B (LimbicB).

**Fig. 3.**
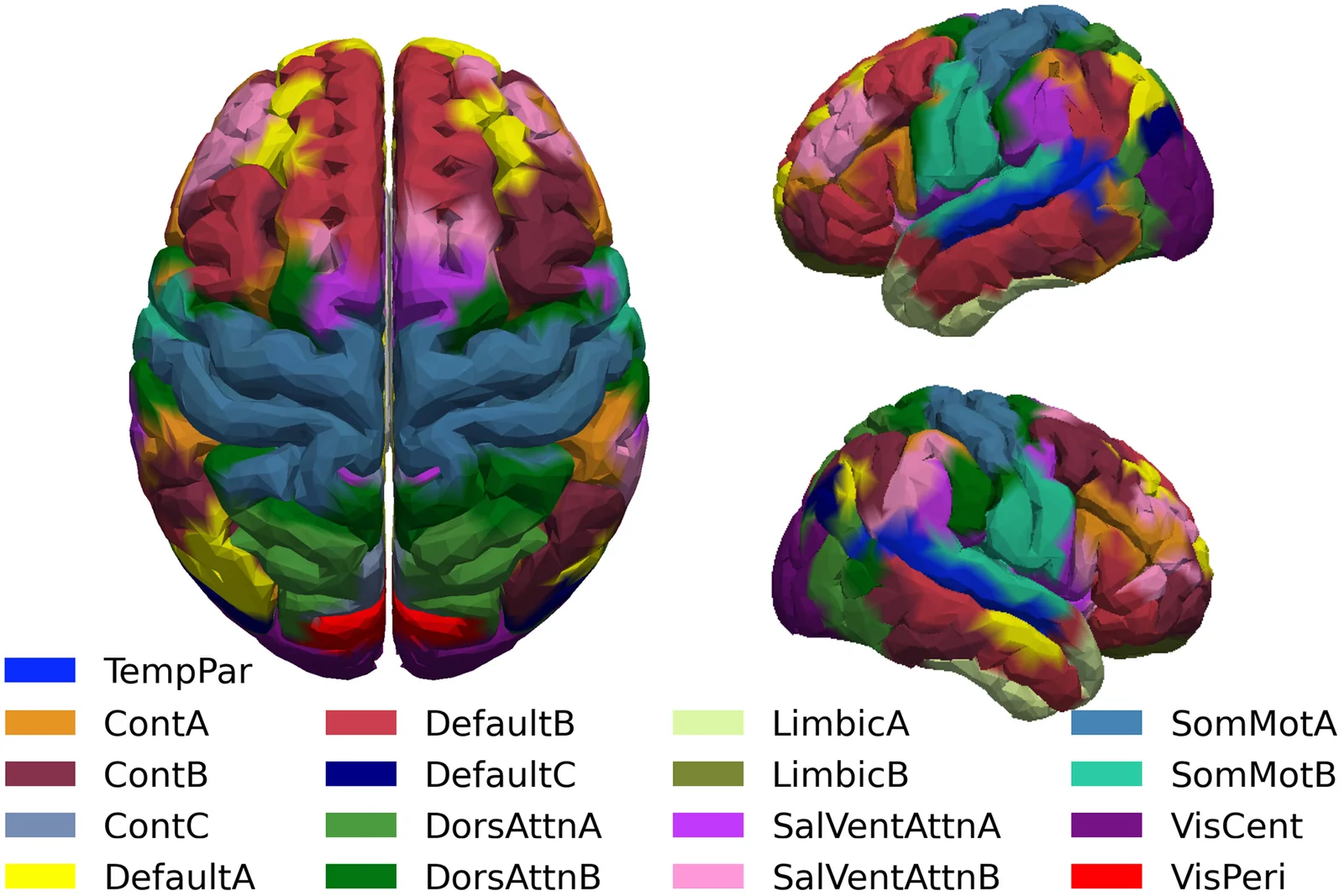
The Schaefer2018 parcellation atlas (Schaefer et al., 2018) with 1000 parcels grouped into 17 functional networks plotted for the cortex of the Colin27 atlas.

### 2.6 Synthetic DOT data

To evaluate the performance of the different head models in source reconstruction, synthetic fNIRS signals, including noise, were generated at the channels of the three probes using the original MRI-based head model. Subsequently, all head models were used to reconstruct the synthetic activity.

#### 2.6.1 Synthetic hemodynamic activity

For each probe separately, we collected the most, median, and least sensitive voxels for each of the 1000 parcels as proxies for that parcel. The median voxel is the typical voxel of a parcel, and its sensitivity matches the parcel’s visible voxel population far better than the most or the least sensitive voxel does. Primarily using the median sensitive voxel, we aim to create a fair source model for comparison between the probe and head models. Figure 4 shows how well a network can be seen across all probe densities and how well the median sensitive voxel sample reflects the network’s sensitivity distribution. Each chosen voxel is used separately as the center of a spatial synthetic hemodynamic response function (HRF). The design of such a 3D synthetic HRF follows a common fMRI approach (Friston et al., 2007): A 3D Gaussian blob of full width at half maximum (FWHM) = 12 mm was placed at the chosen voxel and its neighbors. Its maximum (minimum) amplitude, which is at the center of the Gaussian blob, was set to 15 *μ*M for the HbO concentration (−6 *μ*M for the HbR concentration). This spatial synthetic HRF x was projected with the original (ground truth) forward model A to the different channels y at the scalp surface following Eq. (1).

**Fig. 4.**
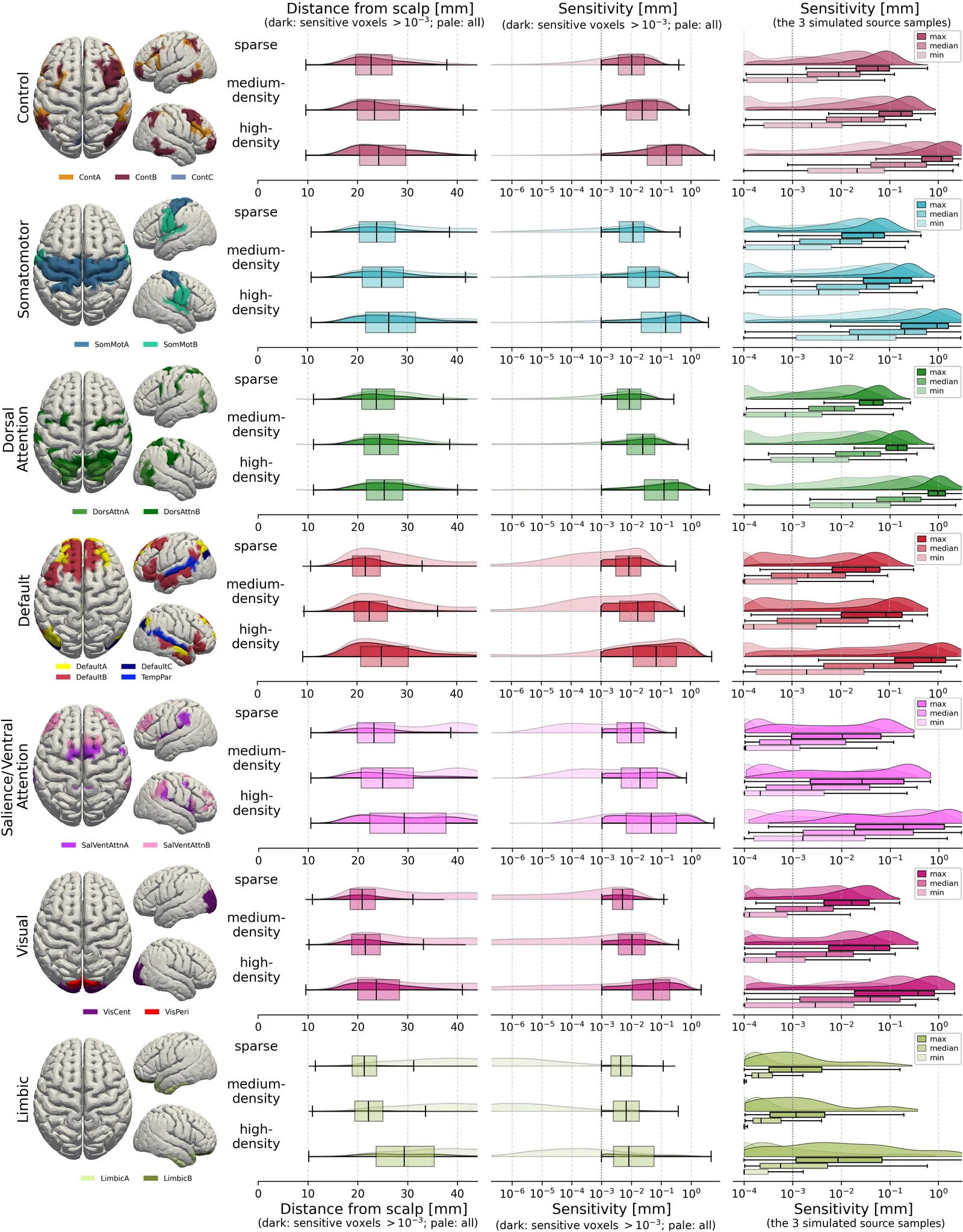
Per coarse Schaefer2018 network (rows) and probe, from the original head model: the network on the cortex (column 1); the scalp distance (column 2) and the sensitivity (column 3) of the sensitive voxels above 10^−3^, each over all network voxels shaded behind; and the most, median, and least sensitive voxel sensitivity distribution samples (column 4, clipped at 10^−4^). Every distribution is drawn as a half violin with a box plot underneath: the box gives the interquartile range, the line inside it the median, and the vertical ticks at either end mark the whiskers at 1.5 times the interquartile range; outliers are not drawn. The three probes, sparse to high density, are stacked top to bottom in every panel.

#### 2.6.2 Measurement noise

While in theory, fNIRS light detection is shot-noise limited, in practice, due to safety limits, sensitivity in modern systems is dominated by measurement noise from the detector electronics. As this noise limits the SNR at longer channels, we include it and model n of Eq. (1) as constant additive white Gaussian noise on all channels. Its normal distribution has zero mean, and its amplitude is scaled to 0.2% of the maximum amplitude of all signals y measured at 30±0.1 mm channels. This corresponds to a signal-to-noise ratio of 54 dB, which is in the typical range of high-performance fNIRS/DOT systems at this channel distance.

### 2.7 Image reconstruction

Finding the inverse operator W to Eq. (1) that solves x=Wy is, in particular in HD-DOT, an ill-posed and generally underdetermined imaging problem (D. Boas et al., 2001). The forward matrix A is reduced to all voxels in either scalp or brain (gray and white matter) tissue, as is done for real-data analysis. Including the scalp allows for capturing potential optode coupling noise and superficial scalp signals (Eggebrecht et al., 2012). Excluding skull and CSF is a reasonable assumption for imaging neural activity. Tikhonov and SVR are commonly applied (Wheelock et al., 2019) to minimize the objective function

$$\underset{\text{x}}{\text{min}}\left\{{\left\|\text{y}-\text{Ax}\right\|}_{2}^{2}+\lambda_{1}^{2}s_{\max}{\left\|\text{Lx}\right\|}_{2}^{2}\right\},$$ (3)

where L is a diagonal matrix with

$$\text{diag}\left(\text{L}\right)=\sqrt{s+\lambda_{2}\max\left(s\right)},\;\;\;\;\;\;s=\text{diag}\left({\text{A}}^{T}\text{A}\right)$$ (4)

following the form written out by Dehghani et al. (2009) and used across high-density DOT (Eggebrecht et al., 2014; Zhan et al., 2012). Brain and scalp voxels are weighted separately, each with its own λ_2_ and its own maximum, so that the two compartments do not compete for the same normalization. Normalizing by max(*s*) makes λ_2_ dimensionless. The Tikhonov regularization parameter λ_1_ balances between image resolution and high-spatial frequency noise. It enters as a penalty of λ_1_ times the largest singular value of $\tilde{\text{A}}$, with *s*_max_ the largest eigenvalue of ${\tilde{\text{A}}}^{T}\tilde{\text{A}}$ , where $\tilde{\text{A}}=\text{A}{\text{L}}^{-1}$ is the SVR-weighted sensitivity matrix, so λ_1_ is dimensionless like λ_2_. The SVR parameter λ_2_ sets how much of the sensitivity range the weighting spans. At λ_2_→0, the penalty on a voxel is proportional to its own sensitivity, so poorly sensitive and typically deeper voxels are lifted. At large λ_2_, the diagonal flattens toward a uniform penalty, which is ordinary Tikhonov regularization without depth weighting. Lower values of λ_2_, therefore, increase the reconstruction amplitude in voxels of low sensitivity, for example, deeper voxels. Forbes et al. (2021) describe the same superficial-bias correction and give the default values used here. λ_2_ runs opposite to the depth-weighting parameters of DOT (Cai et al., 2022; Niu et al, 2010) and of EEG and MEG (Lin et al., 2006), for which larger values mean more compensation. Full depth weighting (a fully column-normalized $\tilde{\text{A}}$) corresponds to λ_2_→0, and no depth weighting to λ_2_→∞. The solution to Eq. (3) is provided by the Moore–Penrose generalized inverse

$$\text{W}={\text{L}}^{-1}{\left({\tilde{\text{A}}}^{T}\tilde{\text{A}}+\lambda_{1}^{2}s_{\text{max}}\text{I}\right)}^{-1}{\tilde{\text{A}}}^{T}\;\text{with}\;\tilde{\text{A}}=\text{A}{\text{L}}^{-1}.$$ (5)

### 2.8 Error computation

To compare source reconstruction results across different head models and subjects, the reconstructed source localizations were transformed from each subject’s individual space to the MNI space. For the MRI-based head models, this was done by using the inverse deformation field as computed during MRI segmentation with SPM (Friston et al., 2007). This inverse MNI deformation field provides the correspondence between the original MRI coordinates and the MNI space. For the photogrammetry-based head models, the same inverse deformation field is given by the individualization methods, while the generic head model Colin27 is by construction already in MNI space. The scaled ICBM-152 needs a backscaling to the original MNI space. All errors reported here are, therefore, computed in MNI space.

The peak localization error is the Euclidean distance between the maximum of the reconstructed activity in the given head model and the maximum of the true synthetic HRF activity in the subject’s true head. In addition to the peak localization error, we report two more metrics. The signed radial error is the component of the peak localization error along the inward cortical normal at the source, and is negative when the reconstruction is pulled toward the scalp. The effective resolution, or 50% containment diameter, combines blob width and displacement into one measure of spatial uncertainty. It is the diameter of the smallest sphere around the true source that still contains every voxel reaching at least 50% of the reconstruction maximum, so it increases when the reconstruction broadens and when it moves away from the source. This is the three-dimensional form of the effective resolution of White and Culver (2010) and Fogarty et al. (2025). Centroid localization error, lateral error, FWHM, and region signal-to-noise ratio were computed as well and are available in the online reference work (Supplementary Section S1, Supplementary Fig. S1) but not reported here, since their results are largely consistent with the metrics above.

## 3 Results

We note that the following results depend on the chosen set of simulation parameters. We provide a quantitative overview within our parameter space to explore the key influences of image reconstruction accuracies that can be expected in different brain regions, depending on the probe design, head model, and regularization parameters. The aim of this work is to draw guidance from these results for a qualitative translation to the broader field, acknowledging that while other probe layouts may, for instance, change the absolute sensitivities to the cortex, qualitative trends and differences between the compared head models will be preserved.

### 3.1 Influence of probe density

For every subject we computed, for each 1 mm isotropic brain voxel of a Schaefer2018 network, its sensitivity to each probe and its distance to the outer scalp surface, both in the subject’s individual space. Figure 4 collects these per network for the original head model: depth and sensitivity of the voxels above 10^−3^, each against all network voxels, and where the three simulated source samples (most, median, and least sensitive voxel of each parcel) fall inside the sensitivity distribution. All 17 networks are shown separately in Supplementary Section S7 (Supplementary Figs. S6–S8) and all 1000 parcels in the online reference work (Supplementary Section S1).

#### 3.1.1 Sensitivity and coverage

Sensitivity rises with probe density and varies strongly across networks. This sorts the seven networks into three coverage classes used throughout this work. Control, Somatomotor and Dorsal Attention are well covered, with a median network sensitivity rising from about $10^{-2.5}$ at the sparse probe to 10^−1^ at the high-density probe and 62 to 92% of their voxels above 10^−3^. Default, Salience/Ventral Attention, and Visual are moderately covered, at $10^{-3.7}$ to $10^{-3.1}$ for the sparse probe and 35 to 80% sensitive across the probes. Limbic is poorly covered: its median sensitivity is $10^{-5.9}$ at the sparse- and $10^{-5.7}$ at the medium-density probes, and it reaches $10^{-3.7}$ , with a third of its voxels above the threshold, only at the high-density probe. Across networks and probes, the sensitive voxels are located 21 to 29 mm below the outer scalp surface, shallowest at the sparse probe and deepest at the high-density probe.

#### 3.1.2 Representativeness of the median sensitivity source

The three source samples tile the sensitivity axis about a decade apart (Fig. 4, fourth column). The median sensitivity voxel used as the source throughout differs by 0.2 decades from the median of the network’s visible voxel population, against 0.7 for the most and 1.5 for the least sensitive voxel. We, therefore, primarily use the median source sample in the following analysis if not otherwise stated (see Supplementary Section S3, Supplementary Fig. S3).

### 3.2 Influence of head models

Figure 5 gives the source localization accuracy of all seven head models, pooled over all 1000 parcels and all 15 subjects, for the sparse-, medium-, and high-density probes. If not otherwise stated, we use the common regularization parameters $\lambda_{1}\;=0.01$ (Tikhonov) and $\lambda_{2}\;=0.1$ (SVR).

**Fig. 5.**
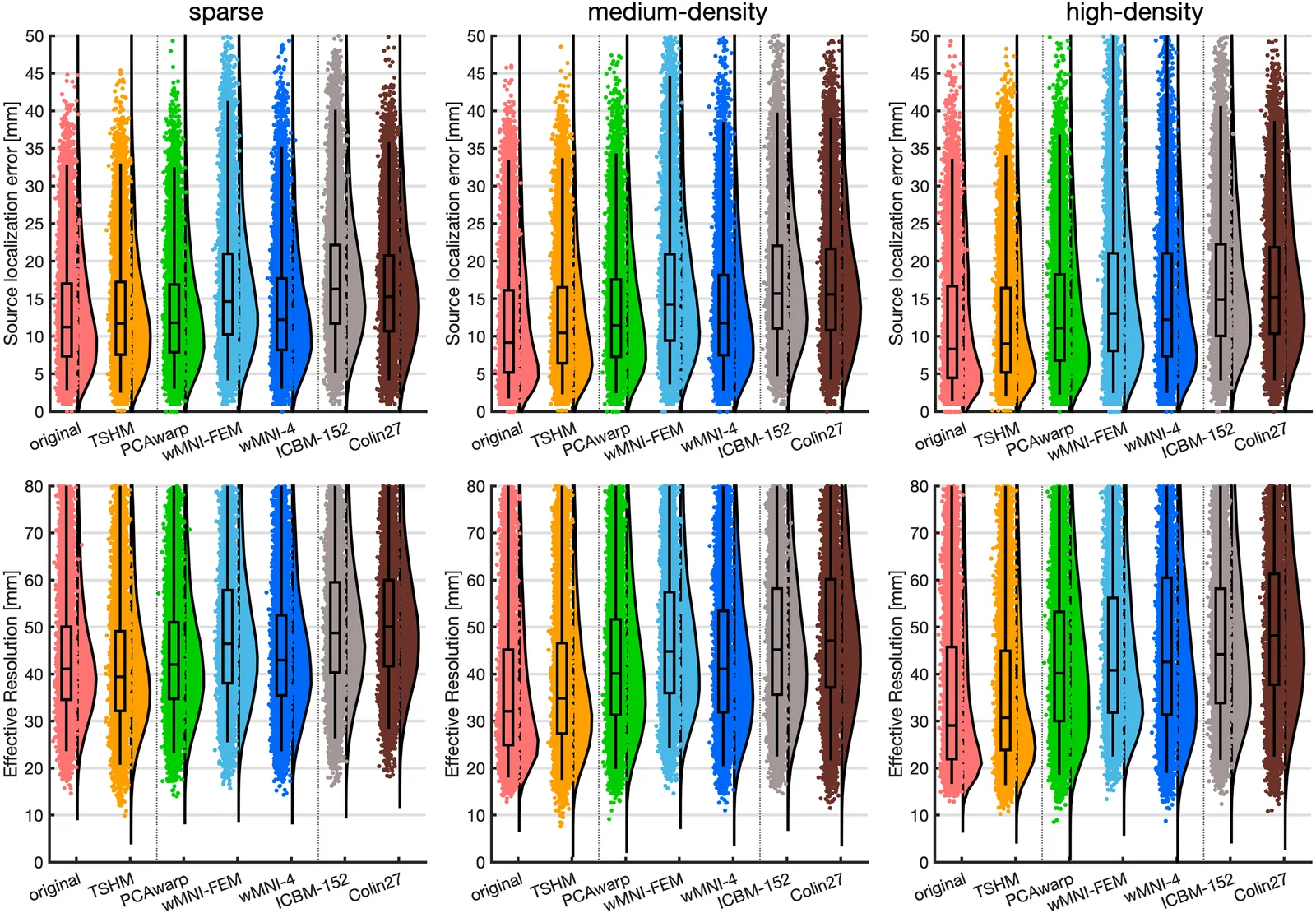
Peak localization error (top row) and effective resolution (bottom row) of the seven head models for the sparse- (left), medium-density (middle), and high-density (right) probes. Effective resolution counts peak displacement as well as blob width (White & Culver, 2010). Each raincloud pools all parcels of all 15 subjects: the dots are the individual parcel results, the half violin is their distribution, and the box shows the median with the interquartile range, its whiskers reaching 1.5 times the interquartile range. One source is simulated per parcel, placed on the voxel with the median sensitivity to the probe; Supplementary Section S3 repeats the comparison for the most and the least sensitive voxel of each parcel. All results include measurement noise and use $\lambda_{1}\;=0.01$ and $\lambda_{2}\;=0.1$ . The MRI-based individual head models are colored pink (original, the ground truth) and orange (TwoSurfaceHeadModel, TSHM), the photogrammetry-based individualized head models green (PCAwarp), light blue (wMNI-FEM), and dark blue (wMNI-4), and the atlas-based generic head models gray (ICBM-152, scaled to landmarks) and brown (Colin27). Dotted lines separate the three groups. Values above the axis limit are not drawn.

#### 3.2.1 Noise

Repeating the identical sources without measurement noise results in the same medians to within a millimeter and slightly narrower distributions (Supplementary Section S2, Supplementary Fig. S2). The head model differences reported here are, therefore, due to the differences in anatomy rather than noise.

#### 3.2.2 MRI-based head models

The original head model is the most accurate across all probes, with median peak localization errors of 11.5, 9.2, and 8.2 mm for the sparse-, medium-, and high-density probes. The error of the TwoSurfaceHeadModel (TSHM) is 0.7 to 1.3 mm larger, significantly so at the medium- and high-density probes. The full gray and white matter geometry of the original head model allows it to leverage the deeper sensitivity provided by a denser probe, whereas the TSHM is restricted to the outer cortical surface. At the high-density probe and with the original MRI, the median peak localization error decreases to 6.6 mm in the well-covered networks and to 2.4 mm on the most sensitive voxel of each parcel (Supplementary Section S3). Ferradal et al. (2014) report a comparable magnitude of 6.6 mm between atlas-based DOT and fMRI peaks in vivo. The extent stays broad even there, with an effective resolution near 29 mm.

#### 3.2.3 Individualized head models

Among the individualized head models, PCAwarp and wMNI-4 are the most accurate with peak localization errors of 11 to 12 mm across the three probes. The two are statistically indistinguishable: across the three probes, the three source samples, and the three metrics, no paired comparison between them reaches significance and no median difference exceeds 0.15 mm. The wMNI-FEM head model, which imposes the cortical folding of the ICBM-152 template on the individual subjects, is worse on peak localization error. Compared with PCAwarp, it localizes 2.2 to 3.0 mm less accurately at every probe, and compared with wMNI-4, it localizes 2.6 to 2.8 mm at the sparse- and medium-density probes. It does, however, have the smallest superficial bias of the three, with a signed radial error 1.8 to 2.1 mm less shallow than PCAwarp at every probe ($p_{\text{Holm}}\;\leq 0.002$ ).

#### 3.2.4 Atlas head models

The two atlas models are the least accurate, with peak localization errors of 15.2 to 16.3 mm. Scaling the ICBM-152 to the subject’s head landmarks does not significantly improve over the unscaled Colin27 at any probe ($p_{\text{Holm}}$ 0.39 to 1.0, *n* = 15).

#### 3.2.5 Effective resolution

The three groups separate on effective resolution only for the denser probes (Fig. 5, bottom row). At the sparse probe, every head model recovers a blob between 39 and 50 mm and the MRI-based models are slightly more accurate, whereas at the high-density probe, the original anatomy sharpens to 28.9 mm and the TSHM to 30.7 mm against 40.1 to 46.9 mm for every model without MRI. Without an MRI, PCAwarp recovers the narrowest blob at every probe, 7.6, 5.1, and 3.1 mm narrower than the better atlas model at the sparse-, medium-, and high-density probes ($p_{\text{Holm}}\;\leq 0.033$ ). The ranking is the same for all three source samples (Supplementary Section S3). The two atlas models recover the widest blob at every probe.

### 3.3 Influence on parcel sensitivity

Figure 6 resolves the errors of Figure 5 over the 1000 Schaefer2018 parcels, grouped by the 7 coarse functional networks and pooled without weighting over parcels and subjects (all 17 networks in Supplementary Section S7, all 1000 parcels in the online reference work, Supplementary Section S1). We compare the networks against the probe coverage shown in Figure 4. The head model ranking of Figure 5 (individual before individualized before atlas) is the same in every network and at every probe except Limbic, where the atlas Colin27 is the lowest model without MRI at the medium- and high-density probes.

**Fig. 6.**
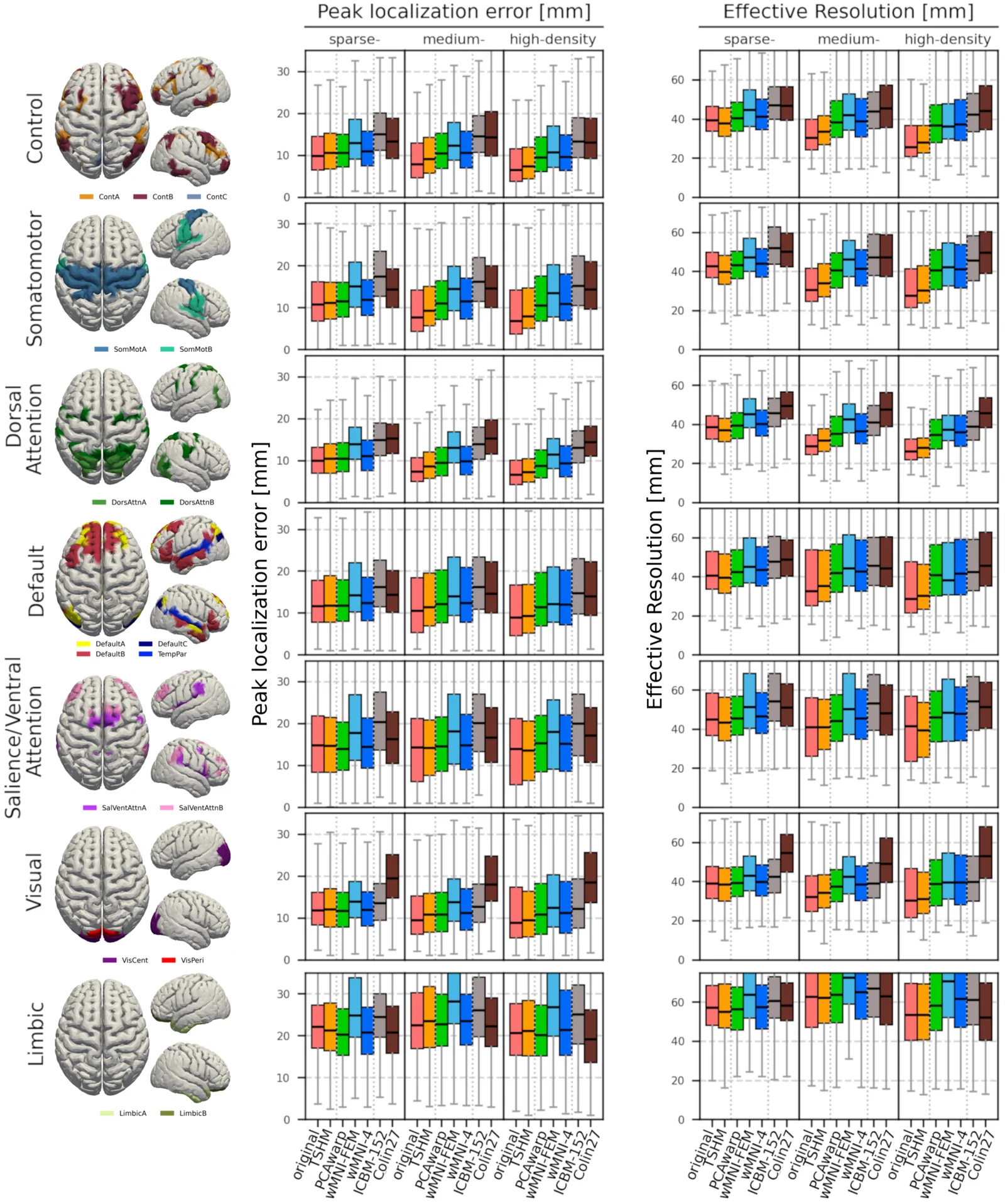
Peak localization error (left block) and effective resolution (right block) per functional network, for the sparse-, medium-density, and high-density probes. Each row is one of the seven coarse Schaefer2018 networks, rendered on the left with its 17-network subdivisions color coded. Boxes give the median and the interquartile range over all parcels of that network and all subjects; whiskers reach 1.5 times the interquartile range, and outliers are not drawn. The y-axes are cut at 35 mm and 75 mm, so the whiskers of the Limbic row are clipped. As in Figure 5, the source is placed on the median-sensitivity voxel of each parcel, measurement noise is included, and $\lambda_{1}\;=0.01$ and $\lambda_{2}\;=0.1$ . Head model colors as in Figure 5. The network-by-network tests behind it are reported in Supplementary Section S4 (Supplementary Tables S1 and S2).

#### 3.3.1 Head models without MRI vs. atlas

The best model without MRI has lower errors than the best atlas across almost every reachable network. On peak localization error, it is significantly better in every network except Limbic at the sparse- and the medium-density probes, and in all but Visual and Limbic at high density, by 1.8 to 4.9 mm ($p_{\text{Holm}}\leq 0.009$ ). On effective resolution, the same holds in every network except Limbic at the sparse probe and in all but Visual and Limbic at medium and high densities, by 2.9 to 7.1 mm ($p_{\text{Holm}}\;\leq 0.037$ ). Across the 21 network-by-probe cells, the best individualized head model is significantly better than the best atlas in 17 cells on localization error and in 16 on effective resolution (Supplementary Tables S1 and S2).

#### 3.3.2 Effective resolution across networks

The reconstruction is a broad blob for every head model. Its effective resolution is about three times the peak localization error at every probe and for every head model (2.4 to 4.1 times across the network and head model cells, Fig. 6, right block), so a reconstruction with a peak error of about 1 cm still spreads over 3 to 4 cm. Effective resolution ranks the head models in the same order as the peak error (individual below individualized below atlas), so blob size and peak accuracy move together. It also shows which changes to the setup sharpen the blob: Increasing the probe density shrinks it by about 10 mm for the individual head models against only 2 to 4 mm for the individualized and atlas models. A denser probe, therefore, sharpens the reconstruction only with an accurate head model anatomy.

#### 3.3.3 Well-covered networks (Control, Somatomotor, Dorsal Attention)

Here the reconstruction is most accurate and most sensitive to the head model. The original head model reaches 10.3, 7.5, and 6.6 mm at the sparse-, medium-, and high-density probes; PCAwarp follows within 1 to 3 mm, and the best atlas model within 3 to 4 mm at 13.7 to 14.7 mm. Probe density improves the MRI-based and individualized models but leaves the atlas models near 14 mm.

#### 3.3.4 Moderately covered networks (Default, Salience/Ventral Attention, Visual)

These networks contain many voxels that no probe can reach (Fig. 4), so the accuracies are bounded. No model, with or without MRI, localizes more accurately than 12 to 13 mm at any probe. At the sparse probe, the individual MRI head models are not outperforming PCAwarp and wMNI-4. In the 17-network split (Supplementary Section S7), the worst is Default C, which is barely visible to any probe. PCAwarp is the best model without MRI, with errors 3.6 to 4.0 mm below the best atlas.

#### 3.3.5 Poorly covered network (Limbic)

Limbic is the least sensitive network to the three probes, so the median peak localization error remains around 20 to 28 mm at the medium- and high-density probes across the MRI-based and individualized head models. The effective resolution ranges from 52 to 73 mm across all models, so the recovered blob is about as wide as the network itself. Colin27 showed the lowest median error without MRI at the medium- and high-density probes, but no model recovers this network at a useful spatial scale.

### 3.4 Influence of SVR

Figure 7 resolves the influence of the SVR parameter λ_2_, for every head model (upper row) and for the ground-truth anatomy alone (remaining rows), along the source sensitivity (computed in the ground-truth head model as used for the synthetic data and plotted in Fig. 4). As derived in Section 2.7, λ_2_ acts as an inverse depth compensation.

**Fig. 7.**
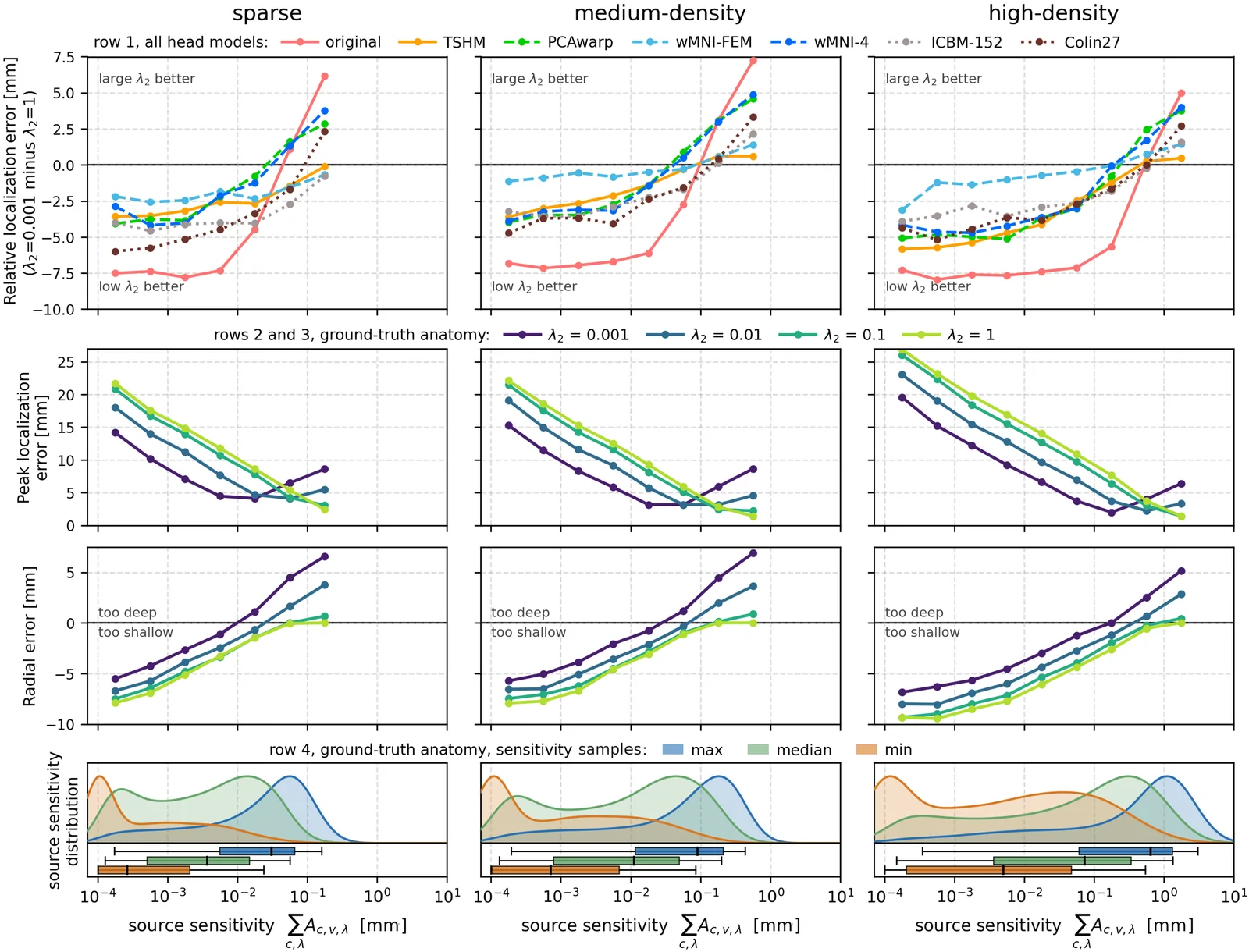
How the SVR parameter λ_2_ acts, resolved along the sensitivity of the simulated source (ground-truth head model) for the sparse- (left), medium-density (middle), and high-density (right) probes. Row 1 gives the effect of λ_2_ for every head model: the peak localization error at the smallest tested value $\lambda_{2}\;=0.001$ minus the error at the largest, λ_2_ = 1. For the ground-truth anatomy only, row 2 shows the peak localization error and row 3 the signed radial error along the inward cortical normal (negative = toward scalp). All three source samples (min, median, and max) are used and row 4 shows their distributions for comparison. Rows 1 to 3 bin the sources by sensitivity in 0.5-decade steps and plot the across-subject median of the per-subject medians in each bin; bins reached by fewer than eight subjects are not drawn. Measurement noise is included and $\lambda_{1}\;=0.01$ . The full four-level λ_2_ sweep for all head models is given in Supplementary Section S5 (Supplementary Fig. S4).

#### 3.4.1 Optimal $\lambda_{2}$ along the sensitivity range

The λ_2_ that minimizes the localization error reverses across the sensitivity range for every head model (Fig. 7, row 1). Below the crossing (lower sensitivity), the small $\lambda_{2}\;=0.001$ reduces the peak localization error by up to 8 mm. Above it (higher sensitivity), the large λ_2_ = 1 reduces it by up to 7 mm. The crossing position is more stable as a rank inside the probe’s own sensitivity distribution than as a sensitivity value, though not fixed. For the ground-truth anatomy, it is at $10^{-1.35}$ , $10^{-1.01}$ , and $10^{-0.27}$ for the sparse-, medium-, and high-density probes, more than a decade apart, but at the 85th, 79th, and 76th percentile of each probe’s own source distribution (row 4) and at the 96th, 92nd, and 88th percentile of all cortex voxels the probe reaches.

#### 3.4.2 Dependence on the head model

The percentile of the crossing also depends on the head model. At medium and high density, it spans the 74th to the 79th percentile for the individual head models, the 62nd to the 77th for the individualized ones, and the 77th to the 87th for the atlases, a total spread of 25 percentile points. A less accurate anatomy, therefore, profits more from the strong depth weighting of a small λ_2_ over a larger part of its range, and only its most sensitive sources gain from raising λ_2_.

#### 3.4.3 Radial localization error

Rows 2 and 3 resolve the radial error behind the results of row 1 for the original MRI head model. Lowering λ_2_ moves the reconstruction deeper by roughly 3 to 5 mm at every source sensitivity. Raising it pulls the reconstruction toward the scalp (row 3, signed radial error). Whether that shift helps depends only on whether the source was reconstructed too shallow in the beginning. At high density, the radial error of the ground-truth anatomy spans − 6.8 to + 5.2 mm at $\lambda_{2}\;=0.001$ and from −9.4 to 0.0 mm at λ_2_ = 1. Therefore, a large λ_2_ corrects the well-sensitive sources that start too deep and over-corrects the poorly sensitive ones past the source toward the scalp. A small λ_2_ corrects the poorly sensitive sources that start too shallow and over-corrects the well-sensitive ones by pulling them too deep.

#### 3.4.4 Cost of a suboptimal $\lambda_{2}$

A suboptimal λ_2_ costs about as much as a suboptimal head model (Fig. 7, row 2). At the high-density probe, the four λ_2_ values span 7.3 mm at the poorly sensitive end of the range and 5.0 mm at the well-sensitive end, against about 7 mm between the best and the worst head model at the same probe. Which value is optimal follows from coverage. Well-covered networks rank high in the probe’s sensitivity distribution, so their optimal λ_2_ rises to the default 0.1 or above as probe density grows, while poorly covered networks rank low and have their lowest error at $\lambda_{2}\;=0.001$ , the Limbic network taking the smallest value in every head model at every probe.

The median-sensitivity sources used throughout this manuscript fall mostly in the lower-coverage part of each probe’s distribution, so at a fixed $\lambda_{1}\;=0.01$ the smallest $\lambda_{2}\;=0.001$ would have given the lower peak localization error in 83% of the network-by-head-model cells, 97% at the sparse probe, and 72% at high density, where the well-covered networks lie above the crossing.

Figures 5 and 6 keep the field-default $\lambda_{2}\;=0.1$ , because the head-model ranking does not change from the smallest to the largest λ_2_ and no head-model conclusion depends on it. The matrix in Figure 8 reports each head model at its own best λ_2_, chosen for each evaluated network, to show every model at its ceiling.

**Fig. 8.**
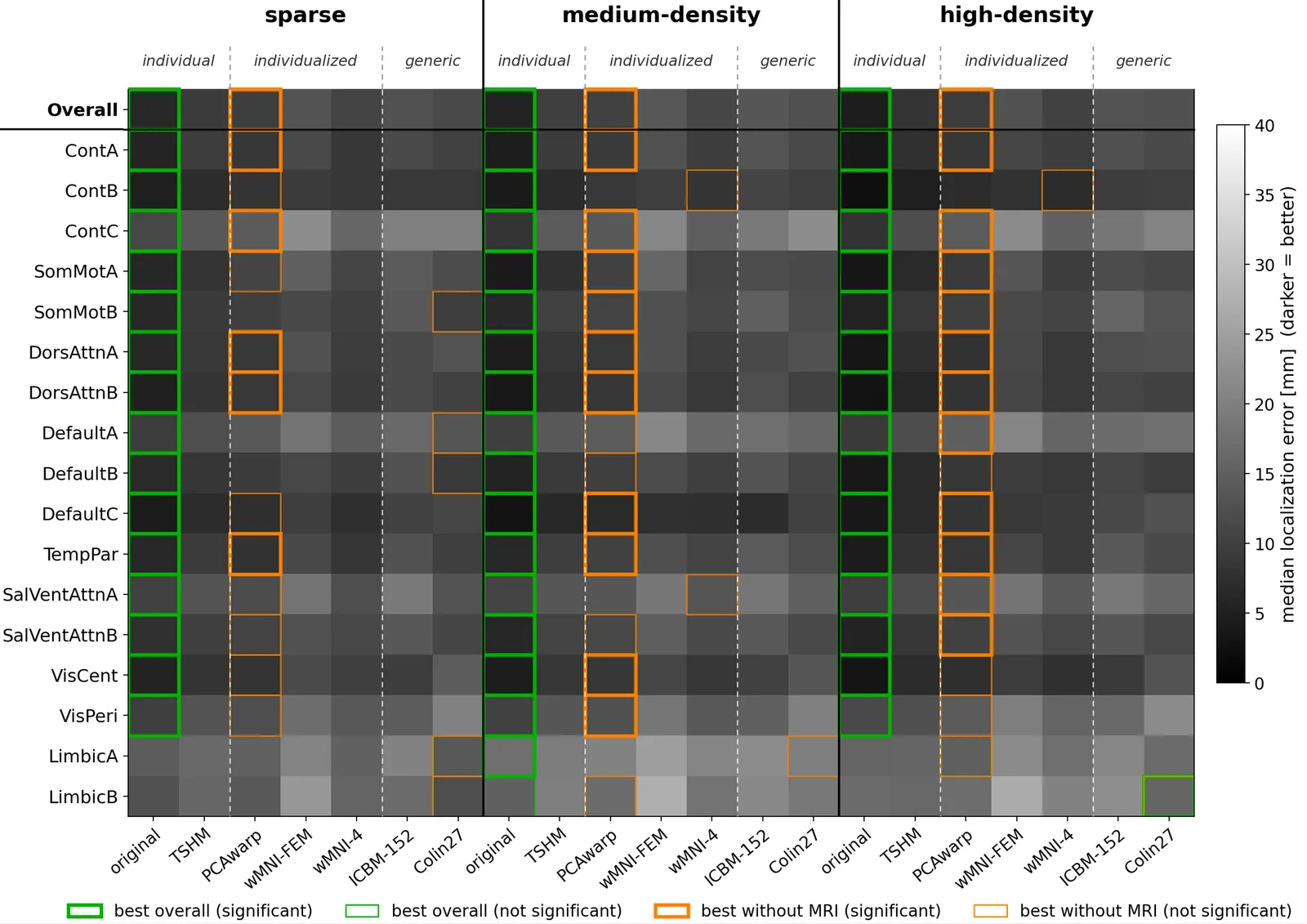
Overview of head model performance per functional network and probe. The grayscale gives the across-subject median peak localization error in millimeters on one global scale (darker = better, clipped at 40 mm), using for each head model individually the most effective of the four tested SVR parameters $\lambda_{2}\;\in \left\{0.001,0.01,0.1,1\right\}$. Rows are the 17 functional Schaefer2018 networks plus an overall row across all parcels, columns the seven head models grouped into individual, individualized, and generic. A green outline marks the best head model overall, an orange outline marks the best head model when no MRI is available. A thick outline marks statistical significance (paired Wilcoxon signed-rank test on the per-subject medians, Benjamini–Hochberg corrected across the grid): green tests the best model overall against the best model without MRI, orange tests the best model without MRI against the best atlas model. The median-sensitivity source sample is used, measurement noise is included, and $\lambda_{1}\;=0.01$ .

The rule of scaling λ_2_ with probe density works only as a side effect. A denser probe places more of its sources above their own crossing, so row 1 keeps its shape across the three columns and only shifts sideways to more sensitive voxels. The choice of λ_2_ thus depends on the rank of the ROI in the probe’s sensitivity distribution (Fig. 4). For sources in the upper part of that distribution, keep λ_2_ at the default 0.1 or higher. For sources in the lower part, lower it toward 0.001, and move the threshold up when working from an atlas head model.

In the full four-level sweep (Supplementary Section S5), the best λ_2_ is significantly better than the default 0.1 in 19 of 21 cells on peak localization error, 11 on effective resolution, and 11 on signed radial error. In 15 of the 21 cells, the λ_2_ that minimizes the peak error differs from the one that minimizes the effective resolution.

### 3.5 Overview of best head model

Figure 8 collects the best head model per network and probe, each model at its own best of the four SVR levels. With a subject MRI, the original head model is the most accurate, with the lowest median error in 50 of the 54 network and probe cells and a significant advantage over the best model without MRI in 49 of them (green outlines). Without an MRI, PCAwarp has the lowest error in 44 of the 54 cells, and in 29 of them the advantage over the best atlas model is significant (orange outlines). Since PCAwarp and wMNI-4 do not differ significantly in any probe, source sample, or metric of the pooled comparison, this cell-by-cell comparison separates the individualized from the atlas models, with PCAwarp and wMNI-4 interchangeable.

In the overall row, PCAwarp localizes 2.1, 2.3, and 3.0 mm more accurately than the best atlas model at the sparse-, medium-, and high-density probes, each at its own best λ_2_ (Benjamini–Hochberg adjusted q=0.026 , 0.0004, and 0.0004).

## 4 Discussion

This study quantified how head model, probe density, and regularization affect the accuracy of HD-DOT source reconstruction, across 15 subjects, 7 head models, 3 probe densities, and 1000 cortical targets, with an emphasis on the common case where no subject MRI is available.

### 4.1 Individualization without an MRI

PCAwarp builds its anatomy from a database of heads, wMNI-4 and wMNI-FEM warp the ICBM-152 template. PCAwarp and wMNI-4 give the lowest peak localization error of any model without MRI at every probe and do not differ from each other; wMNI-FEM localizes 2.2 to 3.0 mm less accurately. The two wMNI models share one warp and differ only in the mesh (smoothed cortex surface against its folded volume), so the 2.6 to 2.8 mm between them at the sparse- and medium-density probes reflects the cost of the imposed folding. These costs shrink as the probe gets denser. In well-covered networks, the individual MRI reaches a median peak error of 10.3 mm at the sparse and 6.6 mm at the high-density probes and the individualized models follow by 1 to 3 mm, close enough that photogrammetry can replace the MRI on peak position when the target ROI is reachable, though not on the extent of the reconstruction. These numbers isolate the anatomy. The same landmark rule places the optodes on every head model, so no model gains or loses from its optode positions. In an MRI-free study, the same photogrammetry scan also provides the individual optode positions, whose absence Srinivasan et al. (2024) identify as a major source of the parcel-sensitivity error of atlas-based models. In practice, a photogrammetry scan, therefore, improves accuracy beyond what we report here.

### 4.2 Atlas head models and coverage

The ICBM-152 scaled to the subject’s head landmarks shows no significant advantage over the unscaled Colin27, and both localize about 4 mm less accurately than the photogrammetry-individualized models at every probe. Scaling to four almost coplanar landmarks or the head circumference matches the scalp in that plane only, whereas the individualization algorithms fit its shape. Cooper et al. (2012) reported an atlas penalty of similar size, 18 mm with Colin27 against 9.1 mm with the subject’s own MRI, where we find 15.8 mm and 9.2 mm at the medium-density probe. Coverage sets a harder limit, because neither a head model nor regularization can recover a network the probe cannot see.

### 4.3 Probe density

With the subject’s own anatomy and the high-density probe, the median peak localization error for the most sensitive voxel of each parcel in the well-covered networks was 2.2 mm in simulation (Supplementary Section S3), below the 6.6 mm DOT-to-fMRI displacement reported in vivo (Ferradal et al., 2014). This agrees with earlier work in which denser arrays brought DOT within reach of fMRI (Eggebrecht et al., 2012; White & Culver, 2010) and spacings of 9.75, 7.5, or 6.5 mm improved source reconstruction further (Fogarty et al., 2025; Habermehl et al., 2012; Markow et al., 2025). In our simulations, a denser probe improved the reconstruction only with an accurate anatomy. For the median source sample, the original head model improved from 11.5 to 8.2 mm from the sparse- to the high-density probes, while the two atlas models stayed between 15.2 and 16.3 mm (Section 3.2).

Both metrics used here favor the denser layouts, but they are only two of several image-quality metrics. Array geometry also varies widely at any given density (Section 2.3), so these numbers transfer to another layout only qualitatively. The extent of the reconstruction stayed broad throughout, with an effective resolution of 20.6 mm even in the best case above.

### 4.4 Spatially variant regularization

The optimal SVR parameter depended on how well the target ROI was covered (Section 3.4). Strong depth compensation (small λ_2_) improved poorly seen targets but overcorrected well-seen ones, which weak compensation (large λ_2_) reconstructed with lower error. The crossing between the two regimes was far more stable as a rank in the probe’s sensitivity distribution than as a fixed sensitivity value, though it still moved by about 25 percentile points across head models. This contrasts with the existence of a depth-invariant optimum reported for the depth compensation algorithm (Niu et al., 2010). We resolved the analysis along sensitivity because the regularizer is built from it and a reader can compute it from their own forward matrix (step 3 of the guideline below). Resolved along sensitivity and across head models, it also quantifies the over-correction that Lin et al. (2006) measured for depth-weighted MEG and that Cai et al. (2022) observed for strong depth weighting in fNIRS. To the best of our knowledge, spatially variant regularization in DOT goes back to Arridge and Schweiger (1993). Pogue et al. (1999) let a scalar regularization parameter vary radially and noted that its functional form is quantitatively similar to $\text{diag}\left({\text{A}}^{T}\text{A}\right)$ of Eq. (4). The operator in the form used here is written out by Dehghani et al. (2009) and inherited across HD-DOT (Eggebrecht et al., 2014; Zhan et al., 2012) without a published derivation or a systematic study of its particular form. The sensitivity-resolved analysis presented here is a first step toward such a study.

### 4.5 Limitations

Several choices bound these results. The synthetic measurements were generated with the sensitivity matrix of the original head model, which is also one of the tested head models, so it is the only head model without a forward-model mismatch. Its advantage over the other head models is, therefore, an upper bound. We also could not separate the reconstruction error from the error of transforming each head model into MNI space (Section 2.8). The effective resolution is outlier sensitive by definition. Its absolute values should not serve as the only resolution metric and should not be compared against FWHM values in the literature. For such comparisons, the online reference work also reports the FWHM (Supplementary Section S1). Supplementary Tables S1 and S2 select the best model without MRI and test it on the same data, which biases them toward individualization. With PCAwarp and the ICBM-152 fixed a priori, the test still finds significant differences in 20 of the 21 network-by-probe cells on peak localization error and in 16 on effective resolution, so the result does not depend on the selection. All 15 subjects are adults, so the infant and early-childhood setting that motivates much of the MRI-free pipeline is untested here. Each parcel is represented by a single voxel, the one at the median of its sensitivity distribution, which is a fair proxy for the typical target but collapses the spread within a parcel to a point. One measurement noise level was simulated, at 54 dB, the level of a high-performance system at a 30 mm channel. At that level, removing the noise changes the medians by less than a millimeter and leaves every ranking unchanged (Supplementary Section S2). Because this study addresses anatomy and regularization, how the head model ranking and the optimal λ_2_ behave at lower SNR or with motion and systemic physiology present remains open. The synthetic HRFs contain no superficial scalp signal, so the analysis does not use the scalp–brain separation that only a dense probe achieves (Eggebrecht et al., 2012; Gao et al., 2023; Yücel et al., 2021), which would further favor denser layouts. Sensitivity decreases with depth, so this design, which resolves the regularization along sensitivity, cannot separate a sensitivity effect from a depth effect. Sex is unevenly sampled, 11 male and 4 female. Since head size and shape differ between the sexes, a more balanced sample could shift the error levels reported here. λ_1_ was fixed at the field default, uncritical over an order of magnitude (Supplementary Section S6, Supplementary Fig. S5), and λ_2_ was swept at that value (Supplementary Section S5), so the two were never optimized jointly.

### 4.6 Limits of the SVR operator

λ_2_ has one degree of freedom, since its exponent is fixed at 1, so it switches the depth weighting on or off without tuning its strength, whereas comparable depth-weighting priors of EEG and MEG expose that exponent as a free parameter (Lin et al., 2006). Adding it as γ and moreover a sensitivity floor α, diag(L)= ${\left(\text{max}\left(s,\alpha \;\text{max}\left(s\right)\right)+\lambda_{2}\text{max}\left(s\right)\right)}^{\gamma /2}$ , did not improve the reconstruction in a small test on five subjects with ground-truth anatomy and the medium-density probe, because no single global setting improved the well-seen and the poorly seen targets at once. The exponent best for deep targets exceeded the one best for shallow targets in every subject, so no single global weight serves both. Because the crossover of Section 3.4 shifts with coverage, the regularization would need to vary with each target’s position in the sensitivity distribution. It would also give the DOT regularization parameter a criterion from the forward matrix alone, where the choice is otherwise made ad hoc, as criticized by Habermehl et al. (2014).

### 4.7 A guideline for reconstruction without an MRI

The findings combine into a short procedure for planning the head model strategy in a DOT study:

**Step 0 Check that the region is reachable** (Fig. 4). Well-covered networks are seen at every density; moderately covered ones need a denser probe, and a poorly covered one like Limbic is not made accurate by any head model or regularization. Change the probe or the question first.

**Step 1 Choose the head model** (Figs. 1 + 8). With a subject MRI, use the individual anatomy. With a photogrammetric scan, use an individualized model. PCAwarp and wMNI-4 perform similarly well, wMNI-FEM localizes 2 to 3 mm less accurately at the sparse- and medium-density probes but shows the smallest superficial bias and improves at high density. With neither MRI nor scan, use an atlas (Colin27 or the scaled ICBM-152). In poorly covered targets, the head models are not distinguishable at all, so improving the reconstruction requires a different probe layout (step 0). Confirm the pick after step 4.

**Step 2 Set** λ_1_ by cross-validation (Habermehl et al., 2014), or take the field default $\lambda_{1}\;=0.01$ . It is uncritical over an order of magnitude (Supplementary Section S6).

**Step 3 Build the forward matrix and plot the sensitivity distribution** over all cortex voxels the probe reaches, and mark where the target ROI falls in it, the way Figure 4 does. Pruning noisy channels before plotting is good practice on real data, although it was not simulated here.

**Step 4 Set the SVR parameter** λ_2_ from the rank of the target in the distribution plotted in step 3 (Fig. 7). On this all-reachable-cortex scale, the crossing for the individual anatomy falls at the 96th, 92nd, and 88th percentile for the sparse-, medium-, and high-density probes. Target ROIs above it recover best at the default $\lambda_{2}\;=0.1$ or above, targets below it at a smaller λ_2_, down to 0.001. The crossing moves down as the probe gets denser and up for a less accurate anatomy (Section 3.4).

**Step 5 Know which accuracy regime you are in** (Figs. 5 + 6), at the default $\lambda_{2}\;=0.1$ : about 1 cm of median peak error with an individual MRI and a dense probe, 1 to 1.5 cm without an MRI, and 1.5 to 2 cm or more with a sparse probe or a poorly covered network. With λ_2_ set per head model as in Figure 8, every regime improves by about 3 mm, to 0.5 to 0.7 cm with an individual MRI, near 1 cm without one and 1.1 to 1.3 cm with an atlas. These values apply to this simulation setup and source model.

The authors are preparing a cedalion Jupyter notebook that follows these steps with one’s own data and probe layout.

### 4.8 Applications of individualized head models

Naturalistic paradigms and stimulus decoding are still reconstructed on atlas head models (Sherafati et al., 2025; Tripathy et al., 2020). Studies of infants and young children, from a few months to 7 years of age and thus across the fastest head growth, rarely have a participant-specific MRI, so they build on atlas-derived or age-matched templates (Collins-Jones et al., 2024; Frijia et al., 2021; Tripathy et al., 2024). Clinical DOT works under the same constraint, for example, when imaging patients with depression during repetitive TMS (Jiang et al., 2021). The gain of an individualized head model should be largest for heads that depart most from the template, which this study cannot show from an adult sample alone. A photogrammetric scan adds a few minutes to the session and closes part of the gap to an individual MRI.

## Supplementary Material

Supplementary Material

## Data Availability

The data that support the findings of this study are available on request from the corresponding author. They are not publicly available due to containing information that could compromise the privacy of research participants. All simulation results are available at http://DOTrecon.de. The code for the individualization algorithms is available at https://github.com/harmening/headmodel_individualization. A practitioner’s guide demonstrates how to perform a photogrammetry scan and how to integrate the scan into the individualization algorithm. The warped head models can be directly exported to cedalion; the authors are also working on including the algorithms into cedalion.

## Footnotes

1
https://www.youtube.com/watch?v=PMBUWHnLXUo

2
https://www.meshlab.net/

## References

[IMAG.a.1445-b1] Aasted, C. M., Yücel, M. A., Cooper, R. J., Dubb, J., Tsuzuki, D., Becerra, L., Petkov, M. P., Borsook, D., Dan, I., & Boas, D. A. (2015). Anatomical guidance for functional near-infrared spectroscopy: AtlasViewer tutorial. Neurophotonics, 2(2), 020801. 10.1117/1.NPh.2.2.020801 PMC4478785 26157991

[IMAG.a.1445-b2] Acar, Z. A., & Makeig, S. (2010). Neuroelectromagnetic forward head modeling toolbox. Journal of Neuroscience Methods, 190(2), 258–270. 10.1016/j.jneumeth.2010.04.031 PMC4126205 20457183

[IMAG.a.1445-b3] Akalin Acar, Z., & Makeig, S. (2013). Effects of forward model errors on EEG source localization. Brain Topography, 26(3), 378–396. 10.1007/s10548-012-0274-6 PMC3683142 23355112

[IMAG.a.1445-b4] Arridge, S. R., & Schweiger, M. (1993). Inverse methods for optical tomography. In H. H. Barrett & A. F. Gmitro (Eds.), Information processing in medical imaging: 13th international conference, IPMI’93, Flagstaff, Arizona (pp. 259–277). Springer. 10.1007/BFb0013793

[IMAG.a.1445-b5] Boas, D., Brooks, D., Miller, E., DiMarzio, C., Kilmer, M., Gaudette, R., & Zhang, Q. (2001). Imaging the body with diffuse optical tomography. IEEE Signal Processing Magazine, 18(6), 57–75. 10.1109/79.962278

[IMAG.a.1445-b6] Boas, D. A., & Dale, A. M. (2005). Simulation study of magnetic resonance imaging-guided cortically constrained diffuse optical tomography of human brain function. Applied Optics, 44(10), 1957–1968. 10.1364/AO.44.001957 15813532

[IMAG.a.1445-b7] Boas, D. A., Dale, A. M., & Franceschini, M. A. (2004). Diffuse optical imaging of brain activation: Approaches to optimizing image sensitivity, resolution, and accuracy. NeuroImage, 23, S275–S288. 10.1016/j.neuroimage.2004.07.011 15501097

[IMAG.a.1445-b8] Cai, Z., Machado, A., Chowdhury, R. A., Spilkin, A., Vincent, T., Aydin, Ü., Pellegrino, G., Lina, J.-M., & Grova, C. (2022). Diffuse optical reconstructions of functional near infrared spectroscopy data using maximum entropy on the mean. Scientific Reports, 12, 2316. 10.1038/s41598-022-06082-1 PMC8831678 35145148

[IMAG.a.1445-b9] Chekin, Y., Decker, D., Dehghani, H., Dubois, J., Field, R. M., Gopalakrishnan, V., Koch, E. M., Lerner, G., Aghajan, Z. M., Miller, N., Olvera, I., Patel, M. J., Perdue, K. L., Schmidt, J., & Szczepanski, V. (2025). A compact time-domain diffuse optical tomography system for cortical neuroimaging. Imaging Neuroscience, 3, imag_a_00475. 10.1162/imagETMaETM00475 PMC12319781 40800861

[IMAG.a.1445-b10] Collins-Jones, L. H., Gossé, L. K., Blanco, B., Bulgarelli, C., Siddiqui, M., Vidal-Rosas, E. E., Duobaitė, N., Nixon-Hill, R. W., Smith, G., Skipper, J., Sargent, T., Powell, S., Everdell, N. L., Jones, E. J. H., & Cooper, R. J. (2024). Whole-head high-density diffuse optical tomography to map infant audio-visual responses to social and non-social stimuli. Imaging Neuroscience, 2, imag-2-00244. 10.1162/imagETMaETM00244 PMC12272203 40800512

[IMAG.a.1445-b11] Cooper, R. J., Caffini, M., Dubb, J., Fang, Q., Custo, A., Tsuzuki, D., Fischl, B., Wells, W., Dan, I., & Boas, D. A. (2012). Validating atlas-guided DOT: A comparison of diffuse optical tomography informed by atlas and subject-specific anatomies. NeuroImage, 62(3), 1999–2006. 10.1016/j.neuroimage.2012.05.031 PMC3408558 22634215

[IMAG.a.1445-b12] Darvas, F., Ermer, J. J., Mosher, J. C., & Leahy, R. M. (2006). Generic head models for atlas-based EEG source analysis. Human Brain Mapping, 27(2), 129–143. 10.1002/hbm.20171 PMC6871464 16037984

[IMAG.a.1445-b13] Dehghani, H., White, B. R., Zeff, B. W., Tizzard, A., & Culver, J. P. (2009). Depth sensitivity and image reconstruction analysis of dense imaging arrays for mapping brain function with diffuse optical tomography. Applied Optics, 48(10), D137. 10.1364/AO.48.00D137 PMC8050954 19340101

[IMAG.a.1445-b14] Eggebrecht, A. T., Ferradal, S. L., Robichaux-Viehoever, A., Hassanpour, M. S., Dehghani, H., Snyder, A. Z., Hershey, T., & Culver, J. P. (2014). Mapping distributed brain function and networks with diffuse optical tomography. Nature Photonics, 8(6), 448–454. 10.1038/nphoton.2014.107 PMC4114252 25083161

[IMAG.a.1445-b15] Eggebrecht, A. T., White, B. R., Ferradal, S. L., Chen, C., Zhan, Y., Snyder, A. Z., Dehghani, H., & Culver, J. P. (2012). A quantitative spatial comparison of high-density diffuse optical tomography and fMRI cortical mapping. NeuroImage, 61(4), 1120–1128. 10.1016/j.neuroimage.2012.01.124 PMC3581336 22330315

[IMAG.a.1445-b16] Fang, Q., & Boas, D. A. (2009). Monte Carlo simulation of photon migration in 3D turbid media accelerated by graphics processing units. Optics Express, 17(22), 20178–20190. 10.1364/OE.17.020178 PMC2863034 19997242

[IMAG.a.1445-b17] Ferradal, S. L., Eggebrecht, A. T., Hassanpour, M., Snyder, A. Z., & Culver, J. P. (2014). Atlas-based head modeling and spatial normalization for high-density diffuse optical tomography: In vivo validation against fMRI. NeuroImage, 85, 117–126. 10.1016/j.neuroimage.2013.03.069 PMC4433751 23578579

[IMAG.a.1445-b18] Fogarty, M., Rafferty, S. M., Markow, Z. E., O’Sullivan, A. C., Svoboda, C. F., George, T., King, K., Wilhelm, D., Tripathy, K., Mugler, E. M., Naufel, S., Yin, A., Trobaugh, J. W., Eggebrecht, A. T., Richter, E. J., & Culver, J. P. (2025). Functional brain mapping using whole-head very high-density diffuse optical tomography. Imaging Neuroscience, *3*, IMAG.a.54. 10.1162/IMAG.a.54 PMC12320020 40800993

[IMAG.a.1445-b19] Fonov, V., Evans, A. C., Botteron, K., Almli, C. R., McKinstry, R. C., Collins, D. L., & Brain Development Cooperative Group. (2011). Unbiased average age-appropriate atlases for pediatric studies. NeuroImage, 54(1), 313–327. 10.1016/j.neuroimage.2010.07.033 PMC2962759 20656036

[IMAG.a.1445-b20] Fonov, V. S., Evans, A. C., McKinstry, R. C., Almli, C. R., & Collins, D. L. (2009). Unbiased nonlinear average age-appropriate brain templates from birth to adulthood. NeuroImage, *47*, S102. 10.1016/S1053-8119(09)70884-5

[IMAG.a.1445-b21] Forbes, S. H., Wijeakumar, S., Eggebrecht, A. T., Magnotta, V. A., & Spencer, J. P. (2021). Processing pipeline for image reconstructed fNIRS analysis using both MRI templates and individual anatomy. Neurophotonics, 8(2), 025010. 10.1117/1.NPh.8.2.025010 PMC8786393 35106319

[IMAG.a.1445-b22] Frijia, E. M., Billing, A., Lloyd-Fox, S., Vidal Rosas, E., Collins-Jones, L., Crespo-Llado, M. M., Perapoch Amadó, M., Austin, T., Edwards, A., Dunne, L., Smith, G., Nixon-Hill, R., Powell, S., Everdell, N. L., & Cooper, R. J. (2021). Functional imaging of the developing brain with wearable high-density diffuse optical tomography: A new benchmark for infant neuroimaging outside the scanner environment. NeuroImage, 225, 117490. 10.1016/j.neuroimage.2020.117490 33157266

[IMAG.a.1445-b23] Friston, K., Ashburner, J., Kiebel, S., Nichols, T., & Penny, W. (Eds.). (2007). Statistical parametric mapping: The analysis of functional brain images. Academic Press. 10.1016/b978-012372560-8/50024-3

[IMAG.a.1445-b24] Gao, Y., Rogers, D., von Lühmann, A., Ortega-Martinez, A., Boas, D. A., & Yücel, M. A. (2023). Short-separation regression incorporated diffuse optical tomography image reconstruction modeling for high-density functional near-infrared spectroscopy. Neurophotonics, 10(2), 025007. 10.1117/1.NPh.10.2.025007 PMC10203730 37228904

[IMAG.a.1445-b25] Habermehl, C., Holtze, S., Steinbrink, J., Koch, S. P., Obrig, H., Mehnert, J., & Schmitz, C. H. (2012). Somatosensory activation of two fingers can be discriminated with ultrahigh-density diffuse optical tomography. NeuroImage, 59(4), 3201–3211. 10.1016/j.neuroimage.2011.11.062 PMC3288812 22155031

[IMAG.a.1445-b26] Habermehl, C., Steinbrink, J., Müller, K.-R., & Haufe, S. (2014). Optimizing the regularization for image reconstruction of cerebral diffuse optical tomography. Journal of Biomedical Optics, 19(9), 096006. 10.1117/1.JBO.19.9.096006 25208243

[IMAG.a.1445-b27] Harmening, N., & Miklody, D. (2022). MRIsegmentation (Version 1.0) [Computer software]. Zenodo. 10.5281/zenodo.7357674

[IMAG.a.1445-b28] Harmening, N., von Lühmann, A., & Blankertz, B. (2026). Data-driven head model individualization from digitized electrode positions or photogrammetry improves M/EEG source localization accuracy. Imaging Neuroscience, 4, IMAG.a.1073. 10.1162/IMAG.a.1073 PMC12784219 41523215

[IMAG.a.1445-b29] Holmes, C., Hoge, R., Collins, L., Woods, R., Toga, A., & Evans, A. (1998). Enhancement of MR images using registration for signal averaging. Journal of Computer Assisted Tomography, 22(2), 324–333. 10.1097/00004728-199803000-00032 9530404

[IMAG.a.1445-b30] Jiang, S., Huang, J., Yang, H., Wagoner, R., Kozel, F. A., Currier, G., & Jiang, H. (2021). Neuroimaging of depression with diffuse optical tomography during repetitive transcranial magnetic stimulation. Scientific Reports, 11, 7595. 10.1038/s41598-021-86751-9 PMC8016845 33795763

[IMAG.a.1445-b31] Lin, F.-H., Witzel, T., Ahlfors, S. P., Stufflebeam, S. M., Belliveau, J. W., & Hämäläinen, M. S. (2006). Assessing and improving the spatial accuracy in MEG source localization by depth-weighted minimum-norm estimates. NeuroImage, 31(1), 160–171. 10.1016/j.neuroimage.2005.11.054 16520063

[IMAG.a.1445-b32] Markow, Z. E., Trobaugh, J. W., Richter, E. J., Tripathy, K., Rafferty, S. M., Svoboda, A. M., Schroeder, M. L., Burns-Yocum, T. M., Bergonzi, K. M., Chevillet, M. A., Mugler, E. M., Eggebrecht, A. T., & Culver, J. P. (2025). Ultra high density imaging arrays in diffuse optical tomography for human brain mapping improve image quality and decoding performance. Scientific Reports, 15(1), 3175. 10.1038/s41598-025-85858-7 PMC11762274 39863633

[IMAG.a.1445-b33] Mazzonetto, I., Castellaro, M., Cooper, R., & Brigadoi, S. (2022). Smartphone-based photogrammetry provides improved localization and registration of scalp-mounted neuroimaging sensors. Scientific Reports, 12, 10862. 10.1038/s41598-022-14458-6 PMC9237074 35760834

[IMAG.a.1445-b34] Middell, E., Carlton, L. B., Moradi, S., Codina, T., Fischer, T., Cutler, J., Kelley, S. M., Behrendt, J., Dissanayake, T., Harmening, N., Yücel, M. A., Boas, D. A., & von Lühmann, A. (2026). Cedalion tutorial: A Python-based framework for comprehensive analysis of multimodal fNIRS and DOT from the lab to the everyday world. Neurophotonics, 13(S3), S32602. 10.1117/1.NPh.13.S3.S32602 PMC13471981 42598192

[IMAG.a.1445-b35] Niu, H., Lin, Z.-J., Tian, F., Dhamne, S., & Liu, H. (2010). Comprehensive investigation of three-dimensional diffuse optical tomography with depth compensation algorithm. Journal of Biomedical Optics, 15(4), 046005. 10.1117/1.3462986 PMC2921418 20799807

[IMAG.a.1445-b36] O’Brien, W. J., Carlton, L., Muhvich, J., Kura, S., Ortega-Martinez, A., Dubb, J., Duwadi, S., Hazen, E., Yücel, M. A., von Lühmann, A., Boas, D. A., & Zimmermann, B. B. (2024). ninjaNIRS: An open hardware solution for wearable whole-head high-density functional near-infrared spectroscopy. Biomedical Optics Express, 15(10), 5625–5644. 10.1364/BOE.531501 PMC11482177 39421779

[IMAG.a.1445-b37] Pogue, B. W., McBride, T. O., Prewitt, J., Österberg, U. L., & Paulsen, K. D. (1999). Spatially variant regularization improves diffuse optical tomography. Applied Optics, 38(13), 2950–2961. 10.1364/AO.38.002950 18319877

[IMAG.a.1445-b38] Schaefer, A., Kong, R., Gordon, E. M., Laumann, T. O., Zuo, X.-N., Holmes, A. J., Eickhoff, S. B., & Yeo, B. T. T. (2018). Local-global parcellation of the human cerebral cortex from intrinsic functional connectivity MRI. Cerebral Cortex, 28(9), 3095–3114. 10.1093/cercor/bhx179 PMC6095216 28981612

[IMAG.a.1445-b39] Scholkmann, F., Kleiser, S., Metz, A. J., Zimmermann, R., Mata Pavia, J., Wolf, U., & Wolf, M. (2014). A review on continuous wave functional near-infrared spectroscopy and imaging instrumentation and methodology. NeuroImage, 85, 6–27. 10.1016/j.neuroimage.2013.05.004 23684868

[IMAG.a.1445-b40] Sherafati, A., Bajracharya, A., Jones, M. S., Speh, E., Munsi, M., Lin, C. H. P., Fishell, A. K., Hershey, T., Eggebrecht, A. T., Culver, J. P., & Peelle, J. E. (2025). A high-density diffuse optical tomography dataset of naturalistic viewing. Scientific Data, 12, 1762. 10.1038/s41597-025-06041-1 PMC12594995 41203640

[IMAG.a.1445-b41] Srinivasan, S., Acharya, D., Butters, E., Collins-Jones, L., Mancini, F., & Bale, G. (2024). Subject-specific information enhances spatial accuracy of high-density diffuse optical tomography. Frontiers in Neuroergonomics, 5, 1283290. 10.3389/fnrgo.2024.1283290 PMC10910052 38444841

[IMAG.a.1445-b42] Tran, A. P., Yan, S., & Fang, Q. (2020). Improving model-based functional near-infrared spectroscopy analysis using mesh-based anatomical and light-transport models. Neurophotonics, 7(1), 015008. 10.1117/1.NPh.7.1.015008 PMC7035879 32118085

[IMAG.a.1445-b43] Tripathy, K., Fogarty, M., Svoboda, A. M., Schroeder, M. L., Rafferty, S. M., Richter, E. J., Tracy, C., Mansfield, P. K., Booth, M., Fishell, A. K., Sherafati, A., Markow, Z. E., Wheelock, M. D., Arbeláez, A. M., Schlaggar, B. L., Smyser, C. D., Eggebrecht, A. T., & Culver, J. P. (2024). Mapping brain function in adults and young children during naturalistic viewing with high-density diffuse optical tomography. Human Brain Mapping, 45(7), e26684. 10.1002/hbm.26684 PMC11069306 38703090

[IMAG.a.1445-b44] Tripathy, K., Markow, Z. E., Fishell, A. K., Sherafati, A., Burns-Yocum, T. M., Schroeder, M. L., Svoboda, A. M., Eggebrecht, A. T., Anastasio, M. A., Schlaggar, B. L., & Culver, J. P. (2020). Decoding visual information from high-density diffuse optical tomography neuroimaging data. NeuroImage, 226, 117516. 10.1016/j.neuroimage.2020.117516 PMC8006181 33137479

[IMAG.a.1445-b45] von Lühmann, A., Kura, S., O’Brien, W. J., Zimmermann, B. B., Duwadi, S., Rogers, D., Anderson, J. E., Farzam, P., Snow, C., Chen, A., Yücel, M. A., Perkins, N., & Boas, D. A. (2024). ninjaCap: A fully customizable and 3D printable headgear for functional near-infrared spectroscopy and electroencephalography brain imaging. Neurophotonics, 11(3), 036601. 10.1117/1.NPh.11.3.036601 PMC11348010 39193445

[IMAG.a.1445-b46] Wheelock, M. D., Culver, J. P., & Eggebrecht, A. T. (2019). High-density diffuse optical tomography for imaging human brain function. Review of Scientific Instruments, 90(5), 051101. 10.1063/1.5086809 PMC6533110 31153254

[IMAG.a.1445-b47] White, B. R., & Culver, J. P. (2010). Quantitative evaluation of high-density diffuse optical tomography: In vivo resolution and mapping performance. Journal of Biomedical Optics, 15(2), 026006. 10.1117/1.3368999 PMC2874047 20459251

[IMAG.a.1445-b48] Yeo, B. T. T., Krienen, F. M., Sepulcre, J., Sabuncu, M. R., Lashkari, D., Hollinshead, M., Roffman, J. L., Smoller, J. W., Zöllei, L., Polimeni, J. R., Fischl, B., Liu, H., & Buckner, R. L. (2011). The organization of the human cerebral cortex estimated by intrinsic functional connectivity. Journal of Neurophysiology, 106(3), 1125–1165. 10.1152/jn.00338.2011 PMC3174820 21653723

[IMAG.a.1445-b49] Yücel, M. A., von Lühmann, A., Scholkmann, F., Gervain, J., Dan, I., Ayaz, H., Boas, D., Cooper, R. J., Culver, J., Elwell, C. E., Eggebrecht, A., Franceschini, M. A., Grova, C., Homae, F., Lesage, F., Obrig, H., Tachtsidis, I., Tak, S., Tong, Y.,… Wolf, M. (2021). Best practices for fNIRS publications. Neurophotonics, 8(1), 012101. 10.1117/1.NPh.8.1.012101 PMC7793571 33442557

[IMAG.a.1445-b50] Zhan, Y., Eggebrecht, A. T., Culver, J. P., & Dehghani, H. (2012). Image quality analysis of high-density diffuse optical tomography incorporating a subject-specific head model. Frontiers in Neuroenergetics, 4, 6. 10.3389/fnene.2012.00006 PMC3359425 22654754

