# Supplementary material for: Improving DOT reconstruction accuracy without MRI: Practical guidelines from systematic head model evaluations
Source: Imaging Neurosci (Camb). 2026 Nov 12;4:IMAG.a.1445. doi: 10.1162/IMAG.a.1445 (PMC13765530; doi:10.1162/IMAG.a.1445)
Supplement: Supplementary file 1 [file IMAG.a.1445_supp.pdf]

Supplementary Material

Improving DOT Reconstruction Accuracy Without MRI: Practical Guidelines from Systematic Head Model Evaluations

Nils Harmening, David A. Boas, Alexander von Lühmann

Figures, tables and sections numbered with the prefix S belong to this Supplementary Material. Numbers without the prefix refer to the main text.

S1 Online reference work

Localization results for all 1000 parcels, 15 subjects, three probes, seven head models, nine metrics, three source samples and all regularization settings are available at http://DOTrecon.de. Fig. S1 shows an exemplary snapshot of the online reference work.

S2 Head model comparison without measurement noise

Fig. 5 of the main text includes measurement noise. Fig. S2 repeats it on the identical synthetic sources with the noise term removed. The medians shift by at most one millimeter and the ordering of the head models is unchanged, so the head model differences in sec. 3.2 originate in the anatomy.

S3 Source samples

Every figure in the main text places the source on the median-sensitivity voxel of each parcel, the voxel that best represents the parcel's visible voxel population (Fig. 4). To show that the head model ranking does not depend on that choice, two additional sources per parcel were simulated on the most and least sensitive voxel to the probe.

Fig. S3 shows that the ranking of the head models is preserved for all three source samples. All errors grow monotonically from the most to the least sensitive source sample. The advantage of individualization over the atlas grows the same way. In Fig. 8, the best model without MRI is significantly better than the best atlas in 15 of the 54 cells for the most sensitive source sample, 29 for the median and 40 for

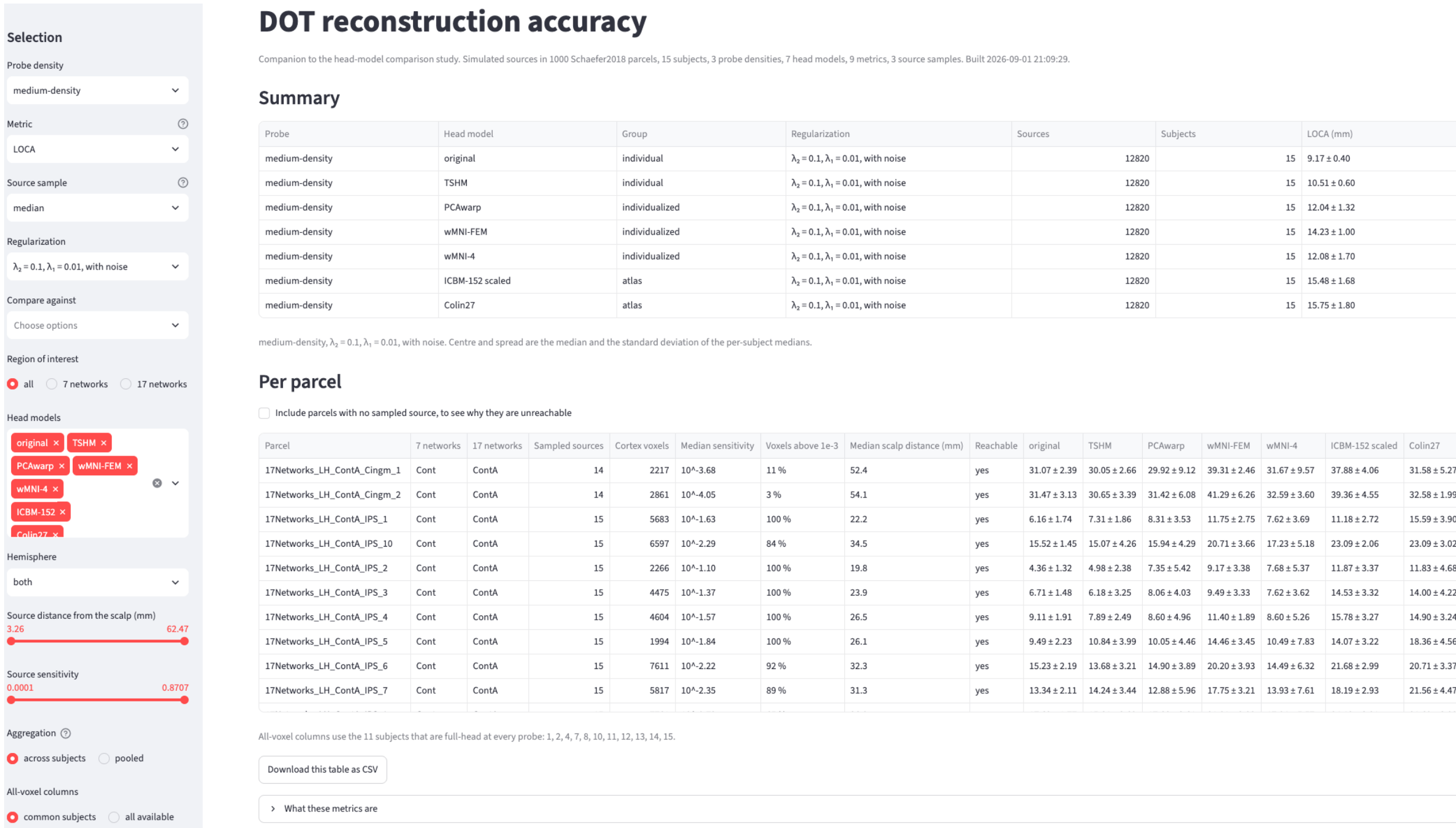

Figure S1: Snapshot of the online reference work in its default view: medium-density probe, peak localization error, median source sample, $\lambda_1 = 0.01$, $\lambda_2 = 0.1$, with measurement noise. The sidebar filters by probe density, metric, source sample, regularization, region of interest (all, 7 or 17 networks), head model, hemisphere, source distance from the scalp, source sensitivity and aggregation (across subjects or pooled). The summary table gives the median and standard deviation of the per-subject medians for each head model. The per-parcel table adds each parcel's networks, number of sampled sources, cortex voxels, median sensitivity, share of voxels above 10^{-3} and median scalp distance, and can be downloaded as CSV.

the least sensitive, while the original head model has the lowest median error in 53, 50 and 36 cells. The median source sample used in the main analysis is therefore a compromise that shows the effect of individualization without overstating it.

The superficial pull also grows as the target becomes less sensitive. For the MRI-based models, PCAwarp and wMNI-4, the fraction of sources reconstructed too shallow rises from 38 to 53 % for the most sensitive source sample to 69 to 81 % for the least sensitive. wMNI-FEM and the two atlas models already reconstruct 60 to 76 % of the most sensitive sources too shallow. From the most to the least sensitive source sample, the median source sensitivity changes by a factor of about 120 and the median distance to the outer scalp surface by 11 to 13 mm. Sensitivity and depth are coupled by construction. Over the sampled sources, the Spearman correlation between the distance to the scalp and the logarithm of the sensitivity is -0.79 to -0.87 across the three probes. Because the three source samples vary sensitivity and depth at once, this comparison cannot separate the two. Fig. 7, row 4, shows the same

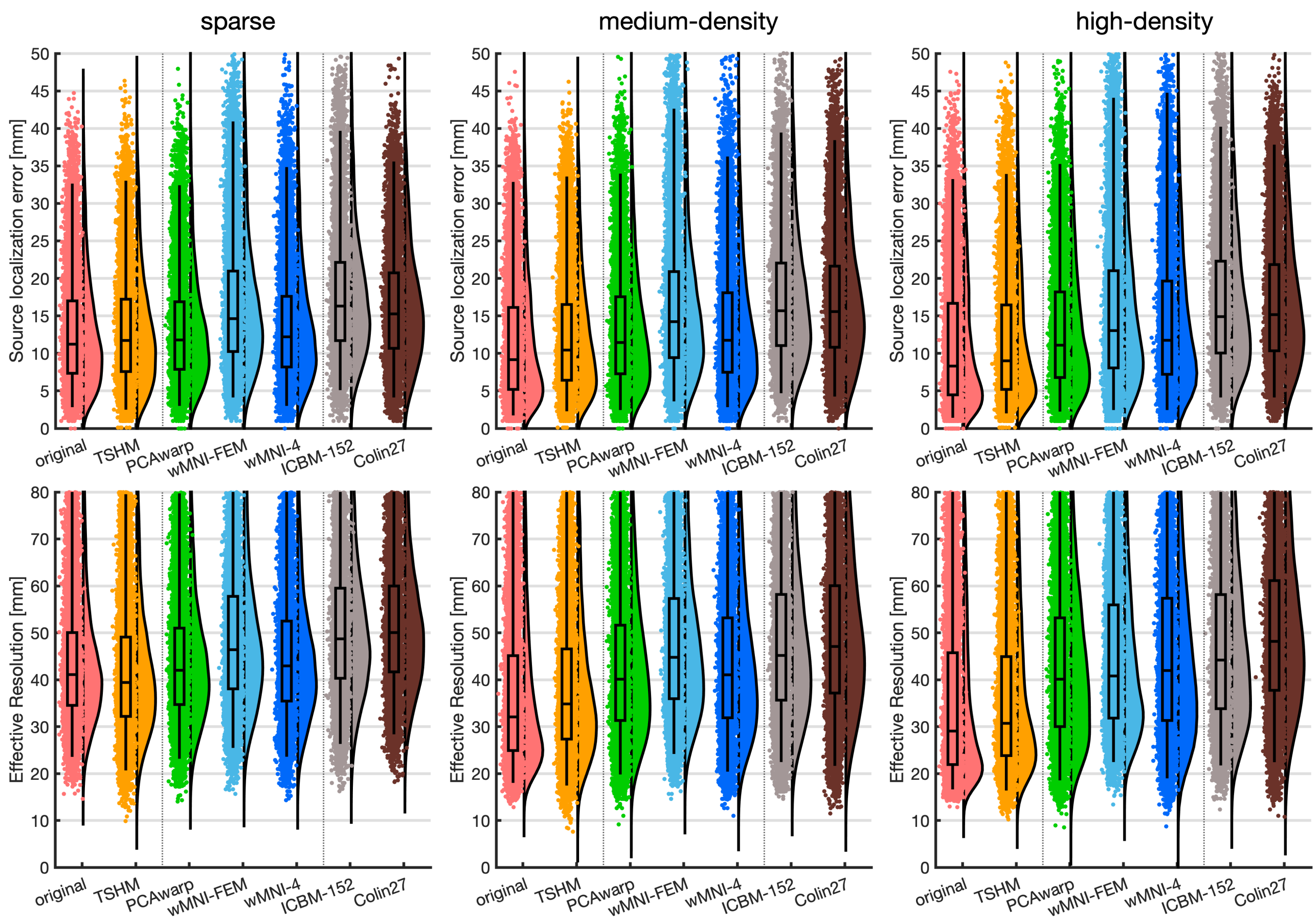

Figure S2: Peak localization error (top row) and effective resolution (bottom row) of the seven head models without measurement noise, for the sparse (left), medium-density (middle) and high-density (right) probe. Same sources, same source model and same regularization as Fig. 5 ($\lambda_1 = 0.01$, $\lambda_2 = 0.1$, median-sensitivity voxel per parcel); only the noise term is removed. Colors and grouping as in Fig. 5. Values above the axis limit are not drawn.

three source samples on the sensitivity scale. All source samples, metrics, head models and regularization settings can be compared in the online reference work (sec. S1).

S4 Network-by-network statistics for Fig. 6

Fig. 6 is descriptive. The tests behind the network-by-network statements of sec. 3.3 are collected here, one row per network and probe, for both metrics. Each row tests whether, without an MRI, the best of the five head models that need no MRI localizes better in this network than the better of the two atlas models a reader would otherwise default to.

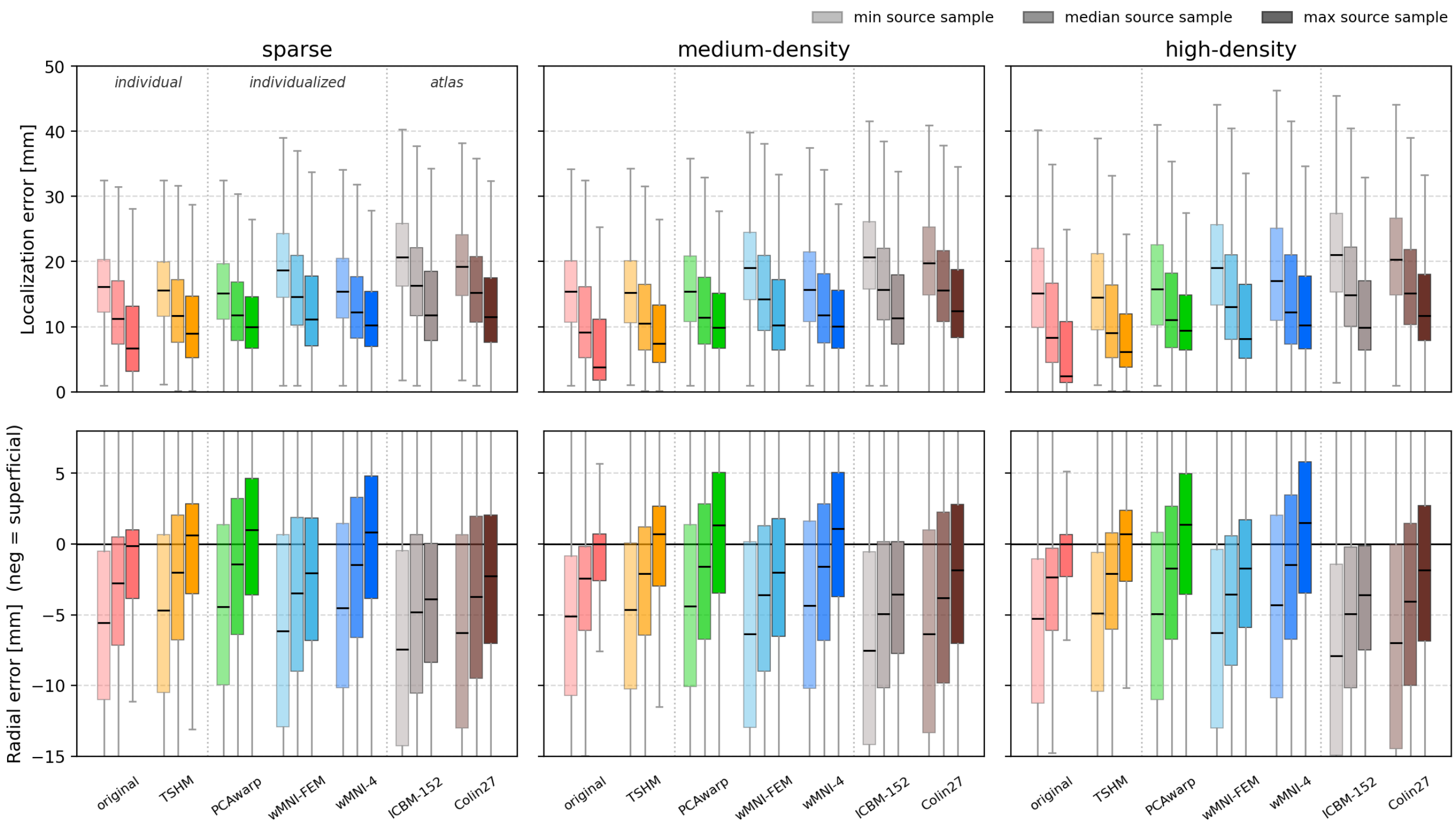

Figure S3: Localization error (top) and signed radial error (bottom) for the three source samples, for the sparse (left), medium-density (middle) and high-density (right) probe. For each parcel, three sources are simulated, on the voxel with the highest, the median and the lowest sensitivity to the probe; the three boxes per head model are the min, median and max source sample from light to solid. Each box gives the median and the interquartile range; whiskers reach 1.5 times the interquartile range, and outliers are not drawn. Colors are as in Fig. 5. Negative radial error means the reconstruction is pulled toward the scalp. For the most sensitive source sample the two individualized models change places with probe density: PCAwarp localizes 0.6 mm more accurately than wMNI-FEM at the sparse probe ($p_{\mathrm{Holm}} = 0.043$), the two do not differ at medium density ($p_{\mathrm{Holm}} = 0.15$), and at the high-density probe wMNI-FEM localizes 1.2 mm more accurately ($p_{\mathrm{Holm}} = 0.002$) and has a 2.9 mm smaller effective resolution ($p_{\mathrm{Holm}} = 0.011$), so the imposed ICBM-152 folding improves localization for the targets the probe sees best. Measurement noise is included, $\lambda_1 = 0.01$ and $\lambda_2 = 0.1$.

probe	network	best without MRI	best atlas	Δ [mm]	r_{rb}	lower	p_{Holm}
sparse	Control	PCAwarp (10.77)	Colin27 (13.11)	-2.36	0.97	14/15	7.3e-04*
sparse	Somatomotor	PCAwarp (11.64)	Colin27 (14.09)	-2.75	0.97	14/15	7.3e-04*
sparse	Dorsal Attention	PCAwarp (10.77)	ICBM-152 (15.07)	-4.09	1.00	15/15	4.3e-04*
sparse	Default	PCAwarp (11.45)	Colin27 (14.04)	-2.74	1.00	15/15	4.3e-04*
sparse	Salience/Ventral Attn.	PCAwarp (13.75)	Colin27 (16.31)	-2.06	0.98	14/15	6.1e-04*
sparse	Visual	PCAwarp (11.53)	ICBM-152 (13.6)	-1.84	0.80	12/15	0.009*
sparse	Limbic	PCAwarp (19.92)	Colin27 (21.21)	-1.55	0.07	9/15	0.847
medium-density	Control	PCAwarp (10.65)	ICBM-152 (13.82)	-4.92	1.00	15/15	4.3e-04*
medium-density	Somatomotor	PCAwarp (11.18)	Colin27 (14.18)	-3.45	1.00	14/15	0.003*
medium-density	Dorsal Attention	PCAwarp (9.49)	ICBM-152 (13.84)	-4.35	1.00	15/15	4.3e-04*
medium-density	Default	PCAwarp (12.85)	Colin27 (14.16)	-2.45	0.95	13/15	0.002*
medium-density	Salience/Ventral Attn.	PCAwarp (14.56)	Colin27 (16.85)	-1.81	0.92	14/15	0.002*
medium-density	Visual	PCAwarp (10.27)	ICBM-152 (11.7)	-2.06	0.83	12/15	0.005*
medium-density	Limbic	Colin27 (21.68)	—	—	—	—	—
high-density	Control	PCAwarp (9.49)	Colin27 (12.81)	-3.47	1.00	15/15	4.3e-04*
high-density	Somatomotor	PCAwarp (10.05)	Colin27 (14.09)	-4.30	0.92	13/15	0.002*
high-density	Dorsal Attention	PCAwarp (9.27)	ICBM-152 (13.08)	-3.72	1.00	15/15	4.3e-04*
high-density	Default	PCAwarp (11.62)	Colin27 (13.69)	-2.09	0.98	14/15	6.1e-04*
high-density	Salience/Ventral Attn.	PCAwarp (15.0)	Colin27 (17.26)	-2.91	0.98	14/15	6.1e-04*
high-density	Visual	PCAwarp (9.43)	ICBM-152 (11.79)	-0.00	0.35	8/15	0.505
high-density	Limbic	Colin27 (19.01)	—	—	—	—	—

Table S1: Network-by-network test behind Fig. 6, peak localization error. For every network and probe, the head model with the lowest across-subject median among the five models that need no MRI (PCAwarp, wMNI-FEM, wMNI-4, ICBM-152, Colin27) is tested against the better of the two atlas models with a paired Wilcoxon signed-rank test on the per-subject medians, Holm-corrected within each probe over the 7 networks. Medians in brackets are in mm. Δ is the across-subject median of the per-subject differences, with negative values indicating that the model without MRI localizes better. It is not the difference of the two medians in brackets and can be near zero while those differ, when the per-subject differences split in sign, as in the high-density Visual row. $r_{\mathrm{rb}} = (R_- - R_+)/(R_- + R_+)$ is the matched-pairs rank-biserial correlation, where R_- and R_+ are the sums of the ranks of the absolute per-subject differences over the subjects with a negative and a positive difference, so $r_{\mathrm{rb}} = 1$ when every subject favors the model without MRI and $r_{\mathrm{rb}} = 0$ when both directions balance. Subjects with zero difference are left out, as in the test. The column lower counts the subjects in which the model without MRI has the smaller error. Rows without an entry are those where the lowest median without MRI is itself an atlas model, so there is no individualization claim to test. * marks $p_{\mathrm{Holm}} < 0.05$.

probe	network	best without MRI	best atlas	Δ [mm]	r_{rb}	lower	p_{Holm}
sparse	Control	PCAwarp (40.0)	Colin27 (46.45)	-6.52	1.00	15/15	4.3e-04*
sparse	Somatomotor	wMNI-4 (43.5)	Colin27 (50.4)	-6.72	0.93	14/15	9.2e-04*
sparse	Dorsal Attention	PCAwarp (39.34)	ICBM-152 (46.0)	-7.05	1.00	15/15	4.3e-04*
sparse	Default	PCAwarp (42.43)	ICBM-152 (48.08)	-5.47	1.00	15/15	4.3e-04*
sparse	Salience/Ventral Attn.	PCAwarp (45.41)	Colin27 (50.24)	-5.98	1.00	15/15	4.3e-04*
sparse	Visual	PCAwarp (39.75)	ICBM-152 (43.36)	-3.37	0.95	13/15	9.2e-04*
sparse	Limbic	PCAwarp (56.35)	Colin27 (60.17)	-1.10	0.22	10/15	0.489
medium-density	Control	PCAwarp (38.47)	ICBM-152 (44.29)	-5.19	0.95	14/15	0.002*
medium-density	Somatomotor	wMNI-4 (40.89)	Colin27 (45.76)	-5.25	0.85	14/15	0.01*
medium-density	Dorsal Attention	PCAwarp (35.21)	ICBM-152 (40.45)	-5.23	0.90	12/15	0.005*
medium-density	Default	PCAwarp (42.24)	Colin27 (43.86)	-2.90	0.75	11/15	0.025*
medium-density	Salience/Ventral Attn.	PCAwarp (43.54)	Colin27 (47.85)	-3.91	0.89	12/15	0.014*
medium-density	Visual	PCAwarp (36.77)	ICBM-152 (38.03)	-3.40	0.58	10/15	0.096
medium-density	Limbic	Colin27 (62.9)	—	—	—	—	—
high-density	Control	wMNI-FEM (35.94)	ICBM-152 (41.95)	-5.81	0.98	14/15	8.5e-04*
high-density	Somatomotor	PCAwarp (39.09)	ICBM-152 (46.0)	-5.19	0.72	10/15	0.037*
high-density	Dorsal Attention	PCAwarp (35.07)	ICBM-152 (38.88)	-4.42	0.83	13/15	0.01*
high-density	Default	wMNI-FEM (38.02)	ICBM-152 (40.94)	-4.76	0.94	13/15	0.009*
high-density	Salience/Ventral Attn.	PCAwarp (45.65)	Colin27 (51.03)	-4.45	0.90	12/15	0.005*
high-density	Visual	PCAwarp (34.76)	ICBM-152 (37.79)	-2.32	0.27	9/15	0.779
high-density	Limbic	Colin27 (52.21)	—	—	—	—	—

Table S2: As Table S1, for effective resolution; negative Δ means a smaller effective resolution without MRI.

S5 The full SVR sweep

Fig. 7 resolves λ_2 along the source sensitivity and contrasts only its smallest and largest value. Fig. S4 gives the complete four-level sweep pooled over all sources. It adds the effective resolution and shows the signed radial error for all seven head models, where Fig. 7 shows it for the ground-truth anatomy only.

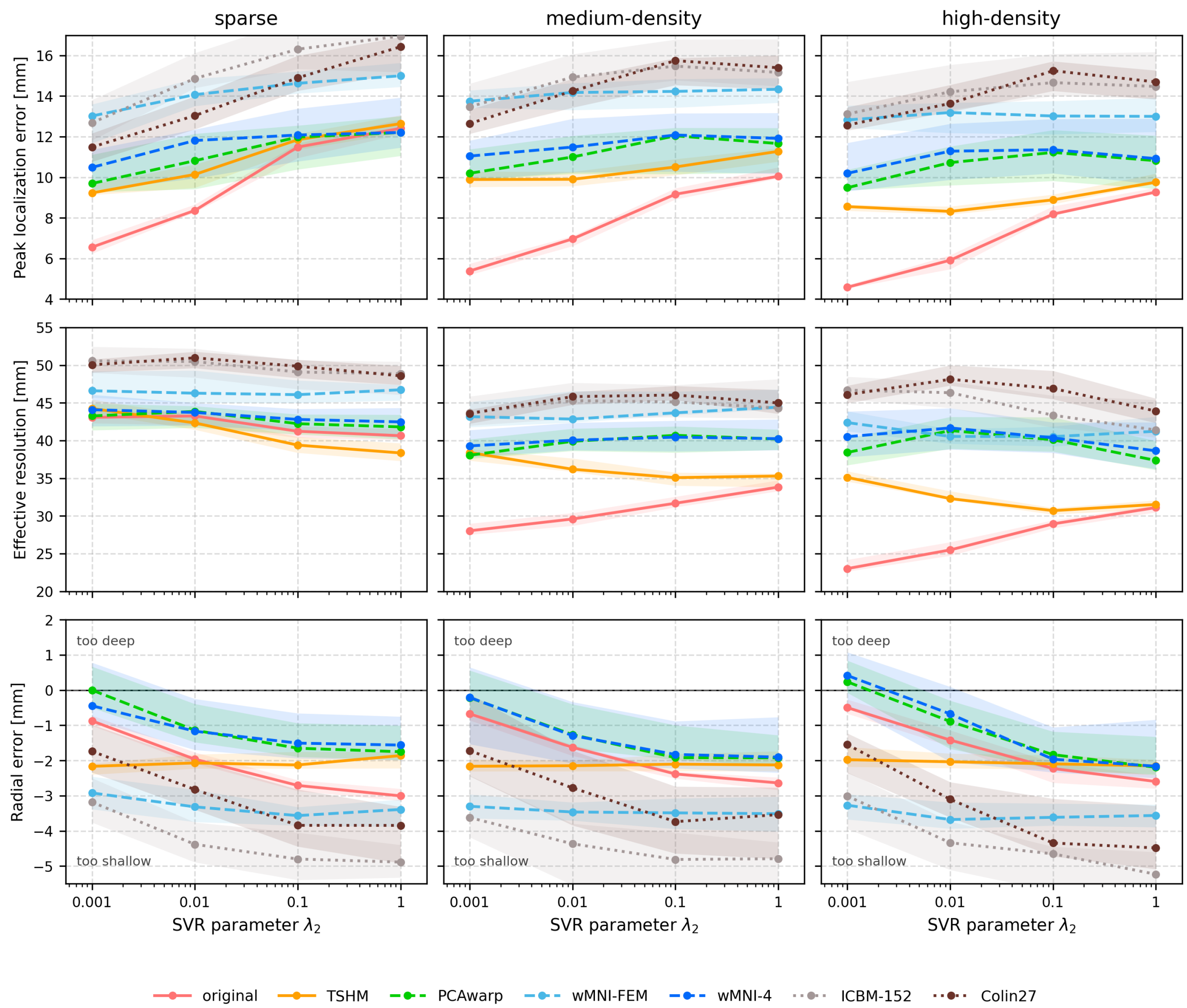

Figure S4: Peak localization error (top), effective resolution (middle) and signed radial error (bottom) of the seven head models over the four tested SVR parameters, for the sparse (left), medium-density (middle) and high-density (right) probe. Lines are across-subject medians and the bands are the across-subject interquartile range. Colors are as in Fig. 5 and the line style repeats the head model grouping (solid individual, dashed individualized, dotted generic). Negative radial error means the reconstruction is pulled toward the scalp. Sources are placed on the median-sensitivity voxel of each parcel; measurement noise is included and $\lambda_1 = 0.01$.

S6 Choice of the Tikhonov parameter

The Tikhonov parameter is fixed at $\lambda_1 = 0.01$ throughout this manuscript, following White and Culver (2010), where this value balanced resolution and contrast-to-noise in in vivo activation data, and Forbes, Wijeakumar, Eggebrecht, Magnotta, and Spencer (2021) recommend it for a broad range of geometries and array designs. Fig. S5 checks the choice against the three other levels that were simulated.

Without regularization, the reconstruction degrades. At $\lambda_1 = 0$ the median peak localization error is worse than at 0.01 in 20 of the 21 head model and probe combinations. It reaches 74 mm for wMNI-FEM and 71 mm for ICBM-152 at the medium-density probe, and 63 and 41 mm for ICBM-152 and the original head model at the high-density probe, with effective resolutions up to 160 mm.

Between 0.001 and 0.1 the curves are flat. Over those three levels, the across-subject median moves by 0.41 mm on the peak localization error and 1.58 mm on the effective resolution, taken as the median over the 21 cells; the worst cell is wMNI-FEM at the sparse probe with 2.05 mm and 5.22 mm. The value 0.01 has the lowest median among the four levels in 9 of 21 cells for peak error and 14 of 21 for effective resolution, and when another nonzero level is lower, it is lower by at most 0.79 mm and 1.43 mm. Testing each head model's best level against 0.01 (paired Wilcoxon on the per-subject medians, Holm-corrected within each probe) yields $p < 0.05$ in 2 of 21 cells for peak error and 3 of 21 for effective resolution. Moving λ_1 by an order of magnitude in either direction therefore changes none of the results reported here.

Two head model and probe combinations deviate from this pattern on peak localization error. At the high-density probe, the TSHM has its lowest error at $\lambda_1 = 0$, 1.7 mm lower in peak localization error and 5.7 mm smaller in effective resolution than at 0.01. We attribute this to the smaller solution space of the TSHM, which is restricted to the cortical surface, but did not test it. On effective resolution, $\lambda_1 = 0$ also gives the lower median at the high-density probe for Colin27, by 2.5 mm, and for PCAwarp, by 0.2 mm. For Colin27 at the sparse probe, $\lambda_1 = 0.1$ gives the marginally lower across-subject median but the higher error in 10 of the 15 individual subjects. For this reason, every test in this manuscript is paired at subject level.

We fix λ_1 rather than selecting it per subject or per head model, although automatic rules exist: Correia, Gibson, Schweiger, and Hebden (2009) compared 11 selection methods on a deblurring and an optical topography problem and concluded that the L-curve is the method of choice, with generalized cross-validation as an alternative for particularly ill-posed problems, and Habermehl, Steinbrink, Müller, and Haufe (2014) proposed cross-validation over the measurement channels for cerebral DOT, which for a linear pseudoinverse such as ours is approximated by the closed-form generalized cross-validation score $g(\lambda) = \|\mathbf{A}\mathbf{A}_\lambda^{\#}\mathbf{y} - \mathbf{y}\|_2^2 \,/\, \mathrm{trace}(\mathbf{I} - \mathbf{A}\mathbf{A}_\lambda^{\#})^2$, evaluated over a grid of λ and minimized, where $\mathbf{A}_\lambda^{\#}$ is the regularized inverse $\mathbf{W}$ of sec. 2.7 at parameter λ. They show that this comes close to the achievable

optimum across a range of noise levels. A per-subject or per-head-model λ_1 would make the head models incomparable here, because any difference between them could then originate in the regularization rather than in the anatomy. Fig. S5 shows that this costs little at the noise level simulated here.

This sweep has two limitations. First, it uses plain Tikhonov without spatially variant regularization, while the SVR inverses behind Fig. 7 are all built at a fixed $\lambda_1 = 0.01$, so the two parameters were never swept jointly. Second, only one measurement noise level was simulated, so the expectation that the optimal λ_1 follows the noise level cannot be tested with these data.

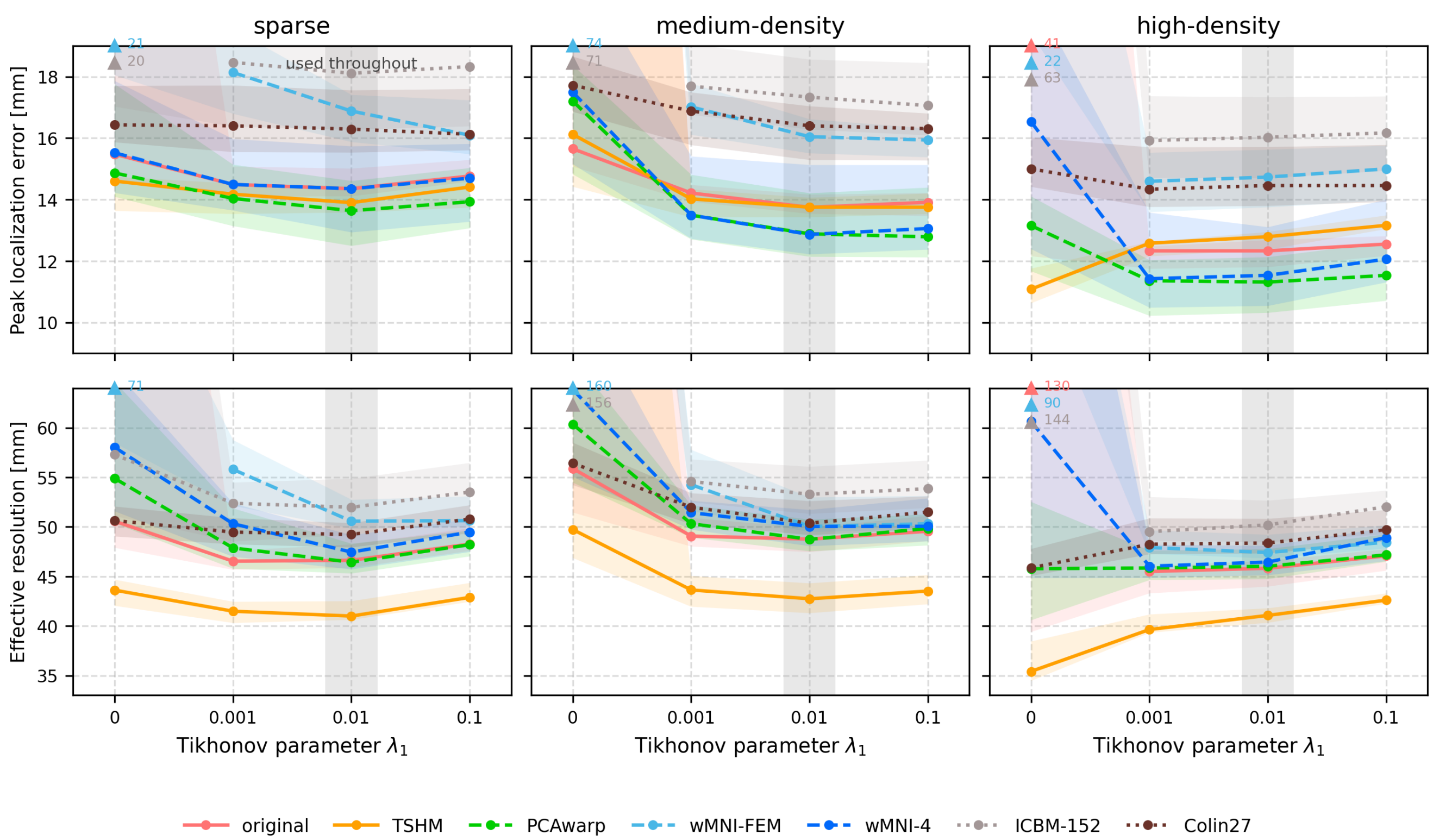

Figure S5: Influence of the Tikhonov parameter λ_1 on the peak localization error (top) and the effective resolution (bottom), for the sparse (left), medium-density (middle) and high-density (right) probe. Lines are across-subject medians and the bands are the across-subject interquartile range. Colors are as in Fig. 5 and the line style repeats the head model grouping (solid individual, dashed individualized, dotted generic). The shaded column marks $\lambda_1 = 0.01$, the value used everywhere else. Medians above the axis limit are drawn as a triangle on the top edge with their value next to it. The x-axis is categorical because $\lambda_1 = 0$ cannot be shown on a logarithmic scale. Sources are placed on the median-sensitivity voxel of each parcel and measurement noise is included. This sweep uses plain Tikhonov without spatially variant regularization.

S7 Results for the 17 functional networks

Figs. S6 to S8 repeat the per-network comparison of Fig. 6 for the 17-network Schaefer2018 subdivision, with the sensitivity distribution of Fig. 4 next to it, so that coverage and reconstruction accuracy appear in one row. Each figure shows one coverage class of sec. 3.3: the well-covered networks, the moderately covered ones, and the poorly covered Limbic network. A denser probe barely reaches Default C. Pooled over all available subjects, the share of its voxels above 10^{-3} rises only from 15.6 to 20.5 % across the three probes, and at the high-density probe it is the smallest of all 17 networks. Individual parcels, the remaining metrics and the other regularization settings can be browsed in the online reference work (sec. S1).

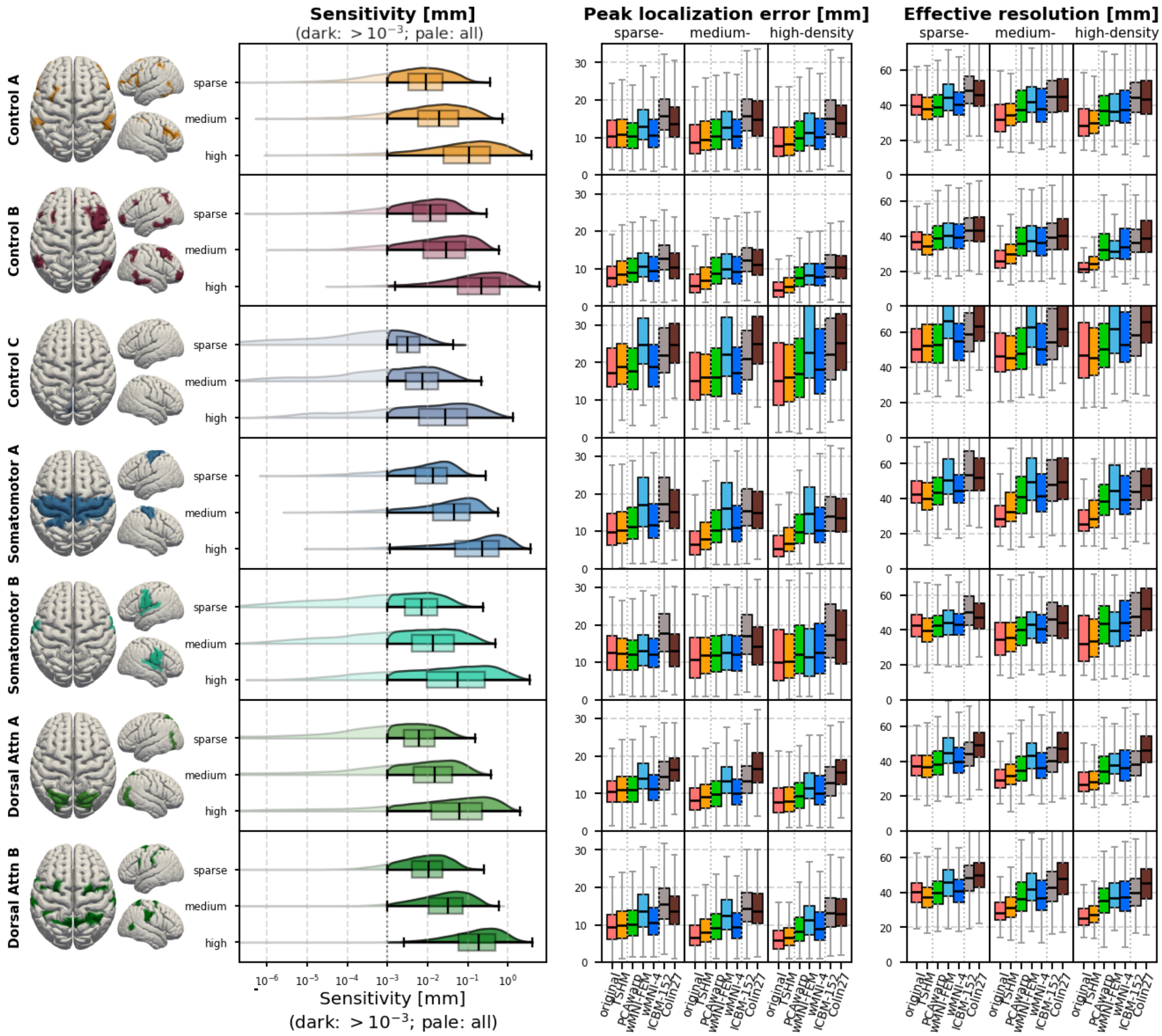

Figure S6: Peak localization error and effective resolution for the well-covered networks of the 17-network Schaefer2018 subdivision. Each row is one network: the network on the cortex, the sensitivity of its voxels with the three probes stacked sparse to high-density (pale: all labeled voxels, dark: those above 10^{-3}, as in Fig. 4), then the peak localization error and the effective resolution for the sparse, medium- and high-density probe. Boxes give the median and the interquartile range over all parcels of that network and all subjects; whiskers reach 1.5 times the interquartile range, and outliers are not drawn. The y-axes are cut at 35 mm and 75 mm as in Fig. 6. Sources are placed on the median-sensitivity voxel of each parcel, measurement noise is included, and $\lambda_1 = 0.01$ and $\lambda_2 = 0.1$. Head model colors as in Fig. 5.

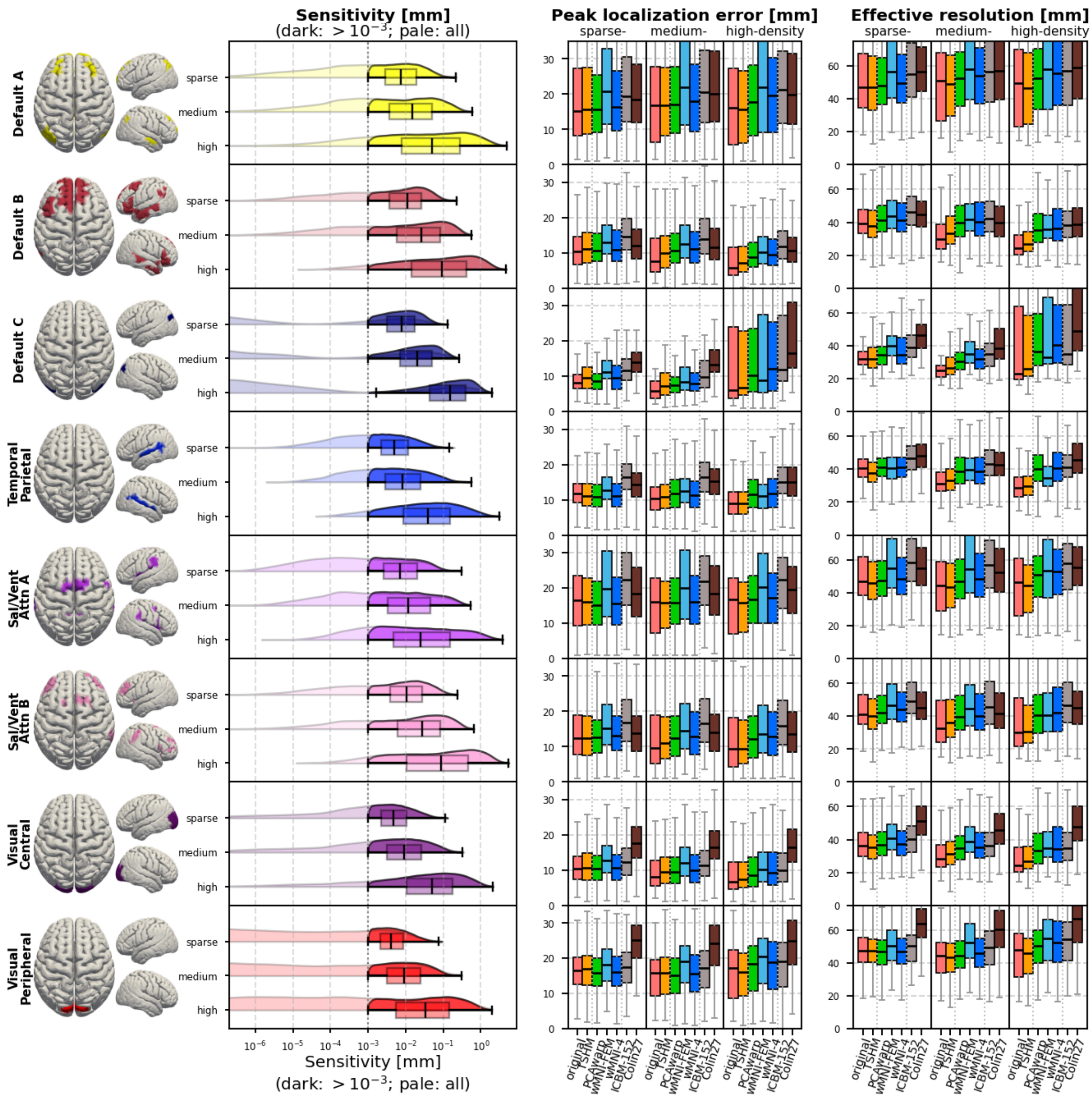

Figure S7: As Fig. S6, for the moderately covered networks (Default, Salience/Ventral Attention, Visual).

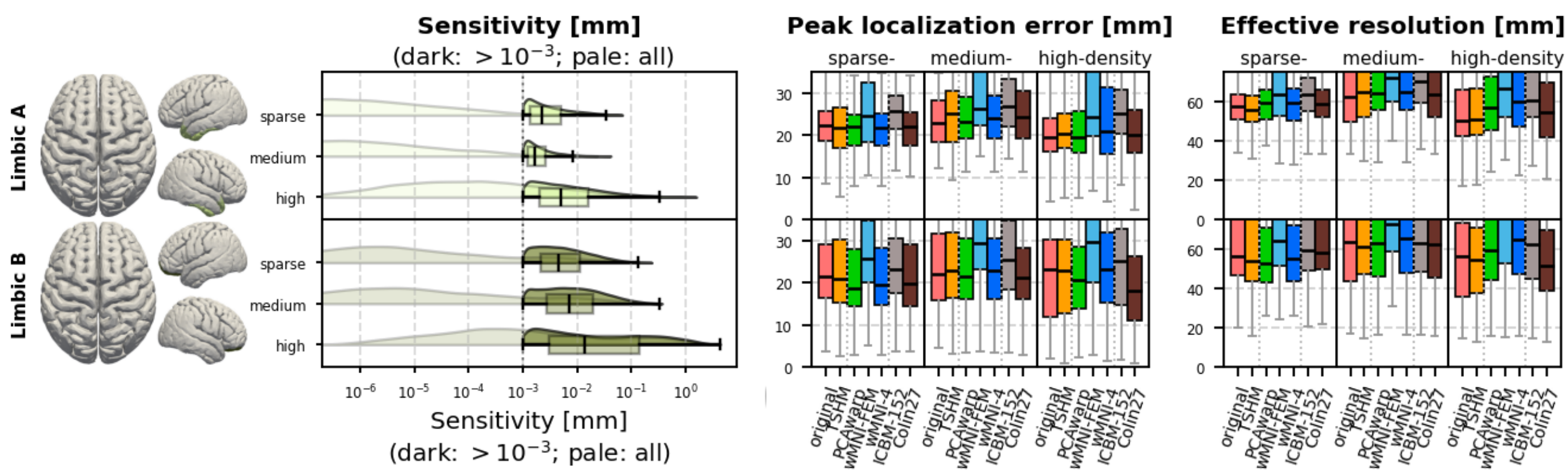

Figure S8: As Fig. S6, for the poorly covered Limbic network.

References

Correia, T., Gibson, A., Schweiger, M., & Hebden, J. (2009). Selection of regularization parameter for optical topography. *Journal of Biomedical Optics*, *14*(3), 034044. https://doi.org/10.1117/1.3156839

Forbes, S. H., Wijeakumar, S., Eggebrecht, A. T., Magnotta, V. A., & Spencer, J. P. (2021). Processing pipeline for image reconstructed fNIRS analysis using both MRI templates and individual anatomy. *Neurophotonics*, *8*(2), 025010. https://doi.org/10.1117/1.NPh.8.2.025010

Habermehl, C., Steinbrink, J., Müller, K.-R., & Haufe, S. (2014). Optimizing the regularization for image reconstruction of cerebral diffuse optical tomography. *Journal of Biomedical Optics*, *19*(9), 096006. https://doi.org/10.1117/1.JBO.19.9.096006

White, B. R., & Culver, J. P. (2010). Quantitative evaluation of high-density diffuse optical tomography: In vivo resolution and mapping performance. *Journal of Biomedical Optics*, *15*(2), 026006. https://doi.org/10.1117/1.3368999
